# SHP1‐Mediated FTO Dephosphorylation Regulates Mitophagy and Promotes Tumor Progression via N(6)‐Methyladenosine in Colorectal Cancer

**DOI:** 10.1002/mco2.71140

**Published:** 2026-11-08

**Authors:** Xi Wang, Qinyu Li, Kai Zeng, Weiheng Zhao, Mu Yang, Sibi Yin, Li Ma, Bo Liu, Hong Qiu, Chaofan Liu, Xianglin Yuan

**Affiliations:** ^1^ Department of Oncology Tongji Hospital Tongji Medical College Huazhong University of Science and Technology Wuhan Hubei China; ^2^ Department of Urology the First Affiliated Hospital of Shihezi University Shihezi Xinjiang China

**Keywords:** colorectal cancer, FTO, N(6)‐methyladenosine, mitophagy, mitochondrial homeostasis, SHP1

## Abstract

Colorectal cancer (CRC) progression and resistance to therapy are driven by complex molecular mechanisms, implying the importance of identifying biomarkers and developing treatment strategies. Whereas Src homology region 2 domain‐containing phosphatase 1 (SHP1) is implicated in immune regulation, its role in tumor malignancy remains unclear. Here, we demonstrate that SHP1 is significantly upregulated in CRC tissues and correlates with poor prognosis. Mechanistically, SHP1 was found to dephosphorylate the N(6)‐methyladenosine (m6A) demethylase fat mass and obesity‐associated protein (FTO) at the Y475 tyrosine residue, thereby enhancing its enzymatic activity to reduce global m6A methylation levels. Integrated RNA sequencing and m6A profiling revealed that SHP1 depletion increased *PTEN‐induced kinase 1 (PINK1) mRNA* m6A modifications, promoting its degradation and suppressing mitophagy. SHP1 inhibitor TPI‐1 or siRNA–SHP1‐loaded lipid nanoparticles (LNPs) attenuated CRC cell proliferation, migration, and tumor growth in xenograft models through impaired mitochondrial homeostasis. These findings establish the SHP1–FTO–m6A axis as a critical regulator of mitophagy‐driven CRC progression. Clinically, our study highlights SHP1 as a prognostic biomarker and potential therapeutic target, with TPI‐1 and LNP–siSHP1 offering novel strategies for CRC management.

## Graphical abstract

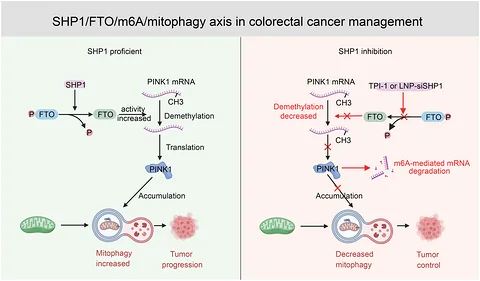

## 1 Introduction

Colorectal cancer (CRC) is one of the most prevalent malignancies worldwide, with an estimated 1.9 million new cases diagnosed in 2020, making it the third most common cancer [1]. Approximately 20% of patients with CRC are initially diagnosed with metastatic disease, which makes most cases incurable [2]. Recent advances in genomic methods have expanded the scope of cancer diagnosis and treatment. Moreover, multiple key genes implicated in the tumorigenesis, metastasis, and therapeutic resistance of CRC have been identified, and therapies targeting these genes have been developed [2, 3]. However, the complex biology of CRC, driven by heterogeneity, immune evasion, or acquired resistance mechanisms ultimately leads to treatment failure [4, 5]. Therefore, there is an urgent need to develop innovative approaches such as new combination regimens, novel immune‐modulating agents, and precision therapies targeting previously untapped pathways to expand therapy strategies.

Src homology region 2 domain‐containing phosphatase 1 (SHP1), also known as protein tyrosine phosphatase nonreceptor type 6 (PTPN6), is a protein tyrosine phosphatase (PTP) that plays an essential role in the negative regulation of signal transduction pathways by dephosphorylating key substrates involved in cell proliferation, differentiation, and survival [6]. Its function is well characterized in hematopoietic cells, where it acts as a critical regulator of immune cell signaling, particularly in controlling cytokine signaling, immune tolerance, and inflammatory responses [7]. However, the role of SHP1 in epithelial cells and cancer cells remains unclear. Emerging evidence suggests that SHP1 may have context‐dependent functions, with its activity shifting from tumor‐suppressive to tumor‐promoting effects in certain cancers [7]. In epithelial cells, the phosphatase activity of SHP1 is believed to influence signaling pathways related to cell adhesion, migration, and angiogenesis, which are critical for tumor progression and metastasis [8, 9]. Our previous study revealed the critical role of SHP1 in CRC radio‐immunotherapy [10]. However, its specific role in colorectal tumor cells remains unclear and requires further investigation.

N(6)‐methyladenosine (m6A), a prevalent internal modification of mammalian mRNA, has emerged as an important mechanism of posttranscriptional gene regulation in CRC. This modification is deposited by methyltransferase complexes containing methyltransferase‐like 3 (METTL3) and methyltransferase‐like 14 (METTL14) and can be removed by demethylases, including fat mass and obesity‐associated protein (FTO) and AlkB homolog 5 RNA demethylase (ALKBH5) [11]. Through interactions with m6A‐associated RNA‐binding proteins, these modifications influence RNA processing, stability, and translation, thereby shaping gene expression programs. In CRC, altered m6A regulation has been implicated in tumor progression, metastasis, and resistance to chemotherapy [12, 13, 14], highlighting its biological and therapeutic relevance. Importantly, the consequences of m6A modification depend on the affected transcripts and their associated regulatory proteins, allowing changes in RNA methylation to produce distinct cellular outcomes. Understanding how upstream signaling pathways regulate m6A‐associated machinery may therefore reveal additional mechanisms underlying CRC progression.

Although m6A‐dependent regulation of PTPN6 transcript stability has been reported in other biological settings [15], whether SHP1 regulates RNA m6A modification through its phosphatase activity in CRC remains unclear. Accordingly, this study aimed to characterize the functions of SHP1 in CRC, investigate its potential role in regulating m6A modification, and determine the contribution of this regulatory pathway to CRC tumor progression. We further evaluated the therapeutic potential of SHP1 inhibition in CRC, which might directly inform the development of new anticancer strategies for CRC management.

## 2 Results

### 2.1 SHP1 is Upregulated in Tumor Cells and Promotes Malignancy in CRC

To investigate the function of SHP1, we first analyzed its expression in CRC. As illustrated in Figure 1A, SHP1 expression was significantly upregulated in tumor tissues compared with adjacent normal tissues based on the tissue microarray HColA160Su02, for which the clinical information is summarized in Table S1. To validate this finding, we examined SHP1 expression in CRC cohorts from The Cancer Genome Atlas (TCGA)‐COAD and Gene Expression Omnibus datasets. As shown in Figures 1B,C and S1A,B, SHP1 was consistently overexpressed in colorectal tumors relative to levels in normal tissues across multiple datasets, and elevated levels were correlated with unfavorable patient outcomes, suggesting its potential role as a prognostic marker for CRC. To further determine whether SHP1 expression independently predicts patient prognosis, we performed multivariate Cox regression analysis incorporating major clinicopathological variables, including gender, T stage, N stage, and M stage. The forest plot showed that high PTPN6 expression remained significantly associated with poor overall survival (Figure S1C). These findings indicate that SHP1 expression is an independent prognostic factor in CRC.

**FIGURE 1:**
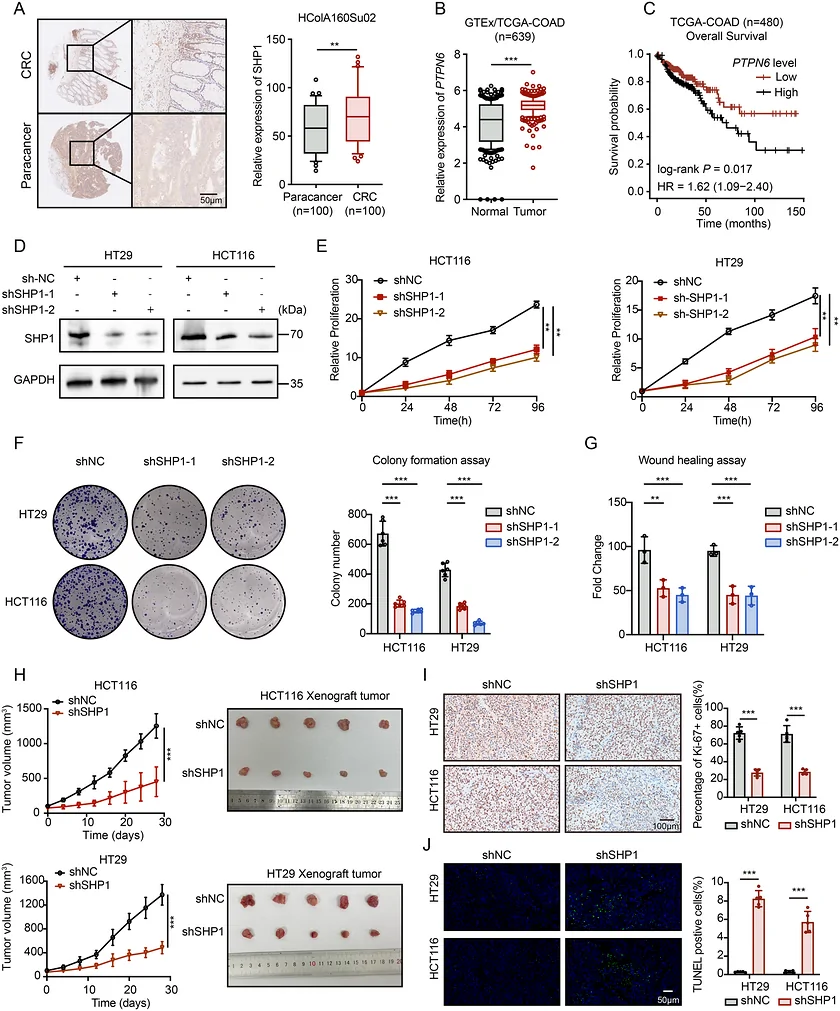
SHP1 is upregulated in CRC and promotes malignant progression. (A) Representative IHC images and quantification of SHP1 expression in CRC and paired paracancerous tissues from the CRC tissue array HColA160Su02. (B) Analysis of SHP1 expression in normal and tumor tissues from the GTEx/TCGA‐COAD dataset. (C) Kaplan–Meier survival analysis of patients with CRC stratified by PTPN6 expression in TCGA‐COAD. (D) Immunoblot validation of SHP1 knockdown efficiency in HT29 and HCT116 cells expressing shNC, shSHP1‐1, or shSHP1‐2. (E) CCK‐8 assays showing reduced proliferation of HCT116 and HT29 cells after SHP1 knockdown with two independent shRNAs. (F) Representative colony formation images and quantification showing decreased clonogenic capacity in SHP1‐depleted HCT116 and HT29 cells. (G) Quantification of wound healing assays showing impaired migratory ability after SHP1 knockdown. (H) Tumor growth curves and representative xenograft tumor images of HCT116 and HT29 cells with or without SHP1 knockdown. (I) Representative Ki‐67 staining images and quantification of Ki‐67‐positive cells in xenograft tumors. (J) Representative TUNEL staining images and quantification of TUNEL‐positive cells in xenograft tumors. Abbreviations: HR, hazard ratio; shRNA, short hairpin RNA, **p < 0.01, ***p < 0.001.

Furthermore, we assessed SHP1 expression across CRC cell lines, including RKO, SW480, HCT116, and HT29, and compared it with that in the normal colonic epithelial cell line NCM460. As presented in Figure S1D,E, both SHP1 mRNA and protein levels were markedly higher in CRC cell lines than in NCM460 cells. To minimize potential off‐target effects, we generated stable SHP1‐knockdown HCT116 and HT29 cells using two independent shRNA sequences, shSHP1‐1 and shSHP1‐2, and confirmed efficient SHP1 depletion by immunoblotting (Figure 1D). Functionally, both shSHP1‐1 and shSHP1‐2 markedly inhibited CRC cell proliferation, as shown by Cell Counting Kit‐8 (CCK‐8) assays and colony formation assays (Figure 1E,F). 5‐ethynyl‐2′‐deoxyuridine (EdU) staining further confirmed that SHP1 knockdown suppressed the proliferative fraction of CRC cells (Figure S2A). Consistently, SHP1 depletion reduced the migratory and invasive abilities of HCT116 and HT29 cells, as validated by wound healing and Transwell assays (Figures 1G and S2B,C). Finally, we evaluated the effect of SHP1 knockdown on tumor growth in vivo using mouse xenograft models. Tumor growth was markedly slower in mice bearing SHP1‐depleted HCT116 or HT29 tumors than in control mice (Figure 1H). Moreover, xenograft tumors derived from SHP1‐depleted cells exhibited reduced staining for the proliferation marker Ki‐67 and increased terminal deoxynucleotidyl transferase dUTP nick‐end labeling (TUNEL) positivity (Figure 1I,J), supporting the role of SHP1 in promoting CRC tumor growth in vivo. Collectively, these findings indicate that SHP1 plays a critical role in promoting malignant behaviors in CRC cells.

### 2.2 SHP1 Interacts with FTO in CRC

SHP1 primarily regulates cellular functions by interacting with its substrates and inducing their dephosphorylation [6]. Therefore, we used immunoprecipitation‐mass spectrometry to identify the SHP1‐binding proteins. As shown in Figure 2A,B, Gene Ontology (GO) analysis of SHP1‐associated proteins revealed an enrichment in biological processes related to mRNA catabolic processes and mRNA stability, indicating a potential role for SHP1 in RNA regulation. Surprisingly, FTO, a pivotal m6A demethylase involved in RNA stability and degradation, ranked high in the GO terms “regulation of mRNA metabolica process” and was selected for further study. We successfully validated the interaction between SHP1 and FTO using co‐immunoprecipitation (co‐IP) assays (Figures 2C,D and S3). To further confirm this interaction, we performed bimolecular fluorescence complementation assays (Figure 2E) and fluorescence colocalization experiments (Figure 2F), both of which supported the physical association between SHP1 and FTO in CRC cells. FTO has an enzymatically active N‐terminal domain (FTO–NT) and a C‐terminal protein–protein interaction domain (FTO–CT) (Figure 2G). By constructing HA‐tagged FTO truncation mutants and subsequent co‐IP assays, we found that SHP1 binds specifically to the FTO–CT (Figure 2H). Taken together, these results demonstrate a specific interaction between SHP1 and FTO in CRC.

**FIGURE 2:**
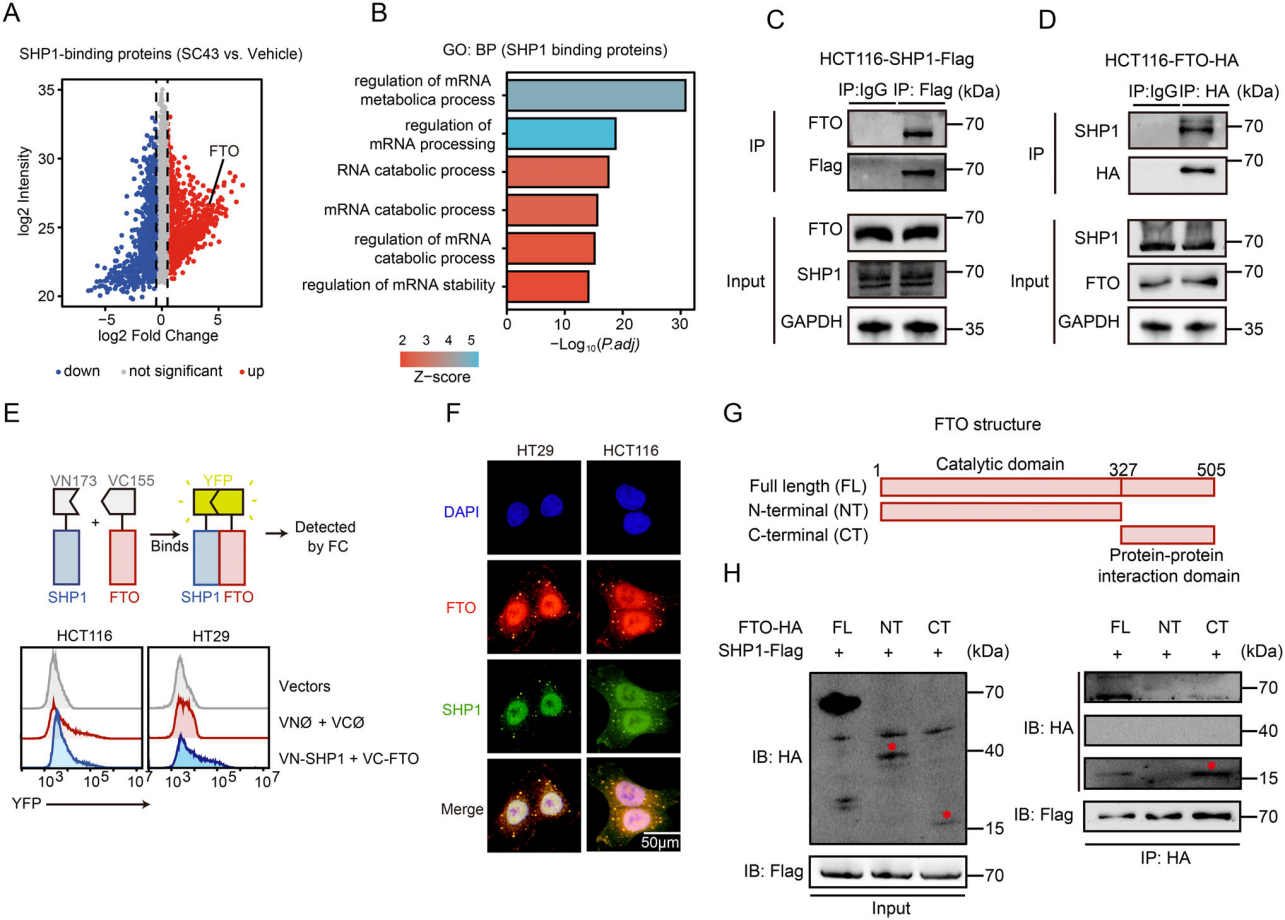
SHP1 interacts with FTO and regulates its activity in CRC cells. (A) Scatter plot showing the interaction between SHP1 and FTO in CRC cells, derived from an immunoprecipitation–mass spectrometry (IP–MS/MS) analysis. (B) GO analysis of SHP1‐associated proteins, indicating the enrichment of mRNA catabolic processes and RNA stability, suggesting a potential role of SHP1 in RNA regulation. (C and D) Co‐IP assays validating the interaction between SHP1 and FTO in HCT116 cells. (E) Bimolecular fluorescence complementation (BiFC) assay demonstrating the interaction between SHP1 and FTO in CRC cells. (F) Immunofluorescence colocalization of SHP1 (green) and FTO (red) in HT29 and HCT116 cells, confirming their physical interaction in the cytoplasm. (G) Diagram showing the domain structure of FTO, with the FTO–NT and FTO–CT domains highlighted. (H) Co‐IP assays using HA‐tagged FTO truncation mutants showing that SHP1 binds specifically to the C‐terminal domain of FTO. Abbreviations: BP, biological process; P.adj, adjusted *p* value; IgG, immunoglobulin G; HA, hemagglutinin epitope tag; FC, flow cytometry.

### 2.3 SHP1 Induces FTO Tyrosine Dephosphorylation at Y475

Next, we explored the effects of the SHP1–FTO interaction. We generated and expressed a substrate‐trapped (enzyme inactive) mutant of SHP1, SHP1–C453S, in HCT116 cells. As shown in Figure 3A, SHP1–C453S exhibited significantly reduced binding to FTO compared with SHP1–WT. This effect was confirmed by using the SHP1 agonist SC43 or its inhibitor TPI‐1 at the concentration within its selective range as previously reported [16]. As shown in Figure 3B, TPI‐1 treatment markedly reduced the binding of SHP1 to FTO. Notably, a sharp decrease in the binding of signal transducer and activator of transcription 3 (STAT3), a consensus substrate of SHP1, was observed, indicating the on‐target effect of TPI‐1. Consistently, SC43 treatment exerted the opposite effects. These results indicated that the interaction between SHP1 and FTO depends on the enzyme activity of SHP1. Therefore, we performed a co‐IP assay to explore the phosphorylation status of FTO following SHP1 knockdown. As shown in Figure 3C, a significant increase in the pan‐tyrosine phosphorylated (pY)‐FTO was observed in shSHP1 cells compared with that in shNC cells. To further confirm the regulatory role of SHP1 in FTO phosphorylation, we utilized a phospho‐assay capable of separating the phosphorylated and unphosphorylated forms of FTO through agarose gel electrophoresis. As shown in Figures 3D,E and S4A, the phosphorylated form of FTO was markedly increased in shSHP1 cells compared with levels in shNC cells. Similarly, TPI‐1 treatment resulted in a concentration‐dependent increase in phosphorylated FTO levels in HCT116 and HT29 cells (Figures 3F,G and S4B). Collectively, these findings suggest that SHP1 promotes FTO dephosphorylation. Next, we used bioinformatics tools to predict potential tyrosine phosphorylation sites on FTO (Figure 3H). Based on this, we generated FTO mutants in which each tyrosine residue was replaced with an alanine to prevent phosphorylation. Among the mutants, only the Y475A mutation suppressed the increase in FTO tyrosine phosphorylation observed upon SHP1 knockdown in HCT116 cells (Figure 3I). Further sequence analysis revealed that Y475 is an evolutionarily conserved site across multiple species (Figure 3J). Collectively, these results indicated that FTO is a major substrate of SHP1 in CRC. SHP1 directly induces FTO dephosphorylation at Y475.

**FIGURE 3:**
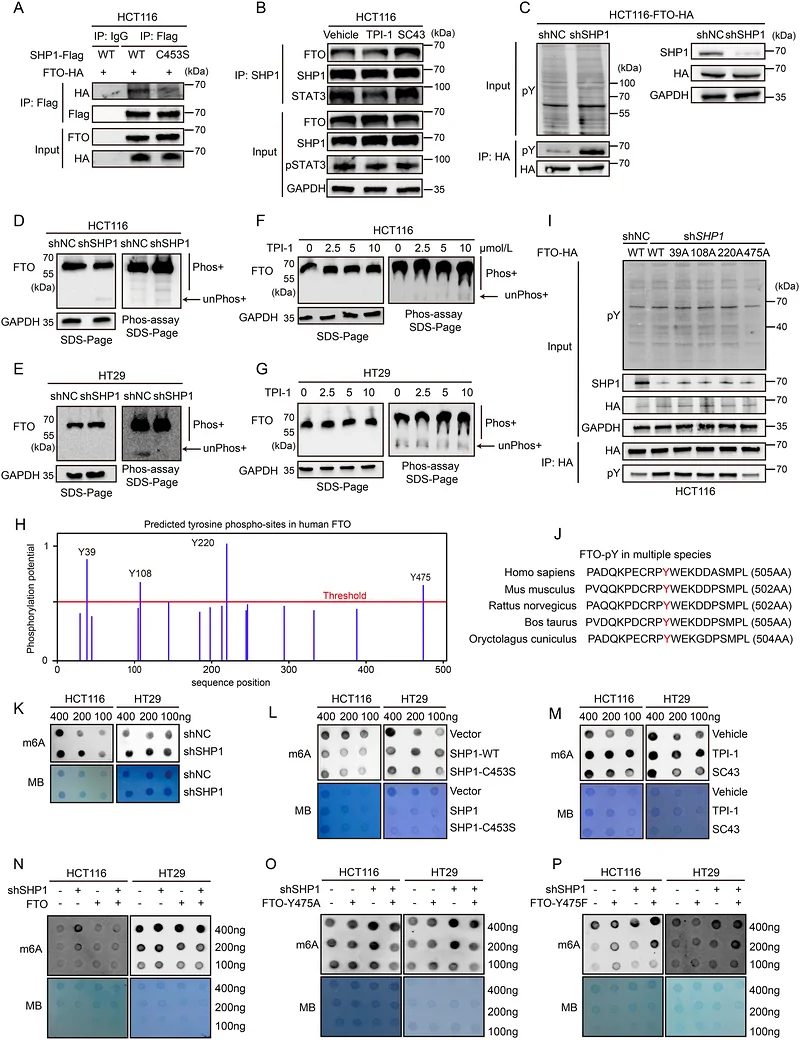
SHP1 dephosphorylates FTO at Y475 and regulates m6A methylation in CRC. (A) Co‐IP assays showing reduced binding between SHP1 and FTO in HCT116 cells expressing the substrate‐trapped SHP1–C453S mutant, compared with that with SHP1–WT. (B) The binding of SHP1 to FTO was assessed in the presence of TPI‐1 (a SHP1 inhibitor) or SC43 (a SHP1 agonist) in HCT116 cells, with pSTAT3 as a substrate control. (C) Co‐IP analysis showing the increased pY of FTO in SHP1‐depleted cells compared with that in control cells. (D and E) Phos‐tag assay to separate phosphorylated and unphosphorylated FTO in SHP1‐depleted cells. (F and G) Phos‐tag assay to separate phosphorylated and unphosphorylated FTO in HCT116 and HT29 cells treated with different doses of TPI‐1. (H) Bioinformatics analysis predicting potential tyrosine phosphorylation sites on FTO, with Y475 identified as a key site. (I) Co‐IP analysis showing that the FTO–Y475A mutation prevents the increase in the tyrosine phosphorylation of FTO upon SHP1 knockdown. (J) Sequence conservation of Y475 across multiple species. (K) m6A dot blot showing increased global m6A methylation in HCT116 and HT29 cells upon SHP1 knockdown. (L) m6A dot blot using HCT116 cells and HT29 cells with wild‐type SHP1, SHP1–C453S mutant, or the control, demonstrating a decrease in m6A with wild‐type SHP1 expression. (M) m6A levels in HCT116 and HT29 cells treated with TPI‐1 or SC43, with TPI‐1 increasing m6A levels and SC43 decreasing these levels. (N) m6A levels in SHP1‐depleted cells overexpressing FTO, showing that FTO overexpression abrogates the increase in m6A caused by SHP1 depletion. (O) m6A dot blot analysis of cells overexpressing FTO–Y475A, which abrogated the m6A increase caused by SHP1 depletion. (P) m6A dot blot analysis of cells expressing the FTO–Y475F mutant, showing enhanced m6A methylation levels compared with those with the control.

### 2.4 SHP1 Regulates m6A Level in CRC via Directly Dephosphorylating FTO at Y475

It was reported that dephosphorylation of FTO–CT regulated its subcellular colocalization and function [17]. Based on the specific regulatory effects of SHP1 on FTO, we hypothesized that SHP1 affects the demethylase activity of FTO. To test this hypothesis, we first conducted m6A dot blot assays to assess global m6A methylation levels. As shown in Figures 3K and S5A, SHP1 knockdown significantly increased m6A methylation in HT29 and HCT116 cells. Similarly, the overexpression of SHP1–WT decreased m6A levels, whereas expression of the inactive mutant SHP1–C453S did not have this effect (Figures 3L and S5B). This evidence was strengthened by using TPI‐1 with HCT116 and HT29 cells, which led to an increase in global m6A levels; in contrast, SC43 reduced the m6A levels (Figures 3M and S5C). To test whether the specific regulatory effect was dependent on FTO–Y475 dephosphorylation, we first overexpressed FTO in shSHP1 cells. As shown in Figures 3N and S6A, FTO overexpression abrogated the increase in m6A caused by SHP1 depletion. Furthermore, the introduction of FTO–Y475A into HCT116 and HT29 cells significantly abrogated the m6A increase mediated by SHP1 knockdown (Figures 3O and S6B), whereas the induction of FTO–Y475F resulted in enhanced m6A methylation compared with that with the vectors (Figures 3P and S6C). Together, these results revealed the functional significance of the SHP1–FTO interaction in CRCs. SHP1 regulates FTO demethylase activity and m6A levels through direct dephosphorylation at Y475.

### 2.5 Integrated Results of RNA‐Seq and meRIP‐Seq Reveal Mitophagy as a Potential Target of the SHP1–FTO Axis

To further elucidate the mechanism through which the SHP1–FTO–m6A axis regulates the biological behavior of CRC, we performed MeRIP‐Seq and bulk RNA‐seq analyses (Figure 4A). As shown in Figure 4B, RNA‐seq analysis revealed a total of 3266 differentially expressed genes (DEGs) between the shSHP1 and shNC cell lines, indicating significant transcriptomic changes after SHP1 knockdown. We further mapped the m6A modification profiles of the two groups. Significantly higher peaks were observed in shSHP1 cells, consistent with the m6A dot blot data (Figure 4C). Notably, the motif analysis of m6A‐enriched regions revealed the GGACU motif as the most enriched motif (Figure 4D), which was previously verified as the most preferable motif of FTO demethylase activity [11]. These results confirm that SHP1 regulates FTO demethylase activity. Next, we depicted the proportion change of m6A sites after SHP1 knockdown, as shown in Figure 4E. Furthermore, we conducted a combined analysis of the DEGs identified from RNA‐seq and the differentially expressed m6A‐modified genes from meRIP‐seq (Figure 4F,G). In total, 309 overlapping genes were identified. Functional enrichment analysis of these genes revealed significant associations with multiple pathways, among which the mitophagy pathway was among the most significantly enriched pathways (Figure 4H). These results were further verified by performing Gene Set Enrichment Analysis, using DEGs from RNA‐seq data and DEGs between SHP1‐low and SHP1‐high groups from TCGA‐COAD (Figures 4I and S7A), with the mitophagy pathway being significantly altered. Similarly, analysis of tumors from single‐cell RNA data revealed higher mitophagy signature in SHP1‐high cells (Figure S7B–D). Taken together, these findings demonstrate that the mitophagy pathway is a potential target of the SHP1–FTO regulatory axis.

**FIGURE 4:**
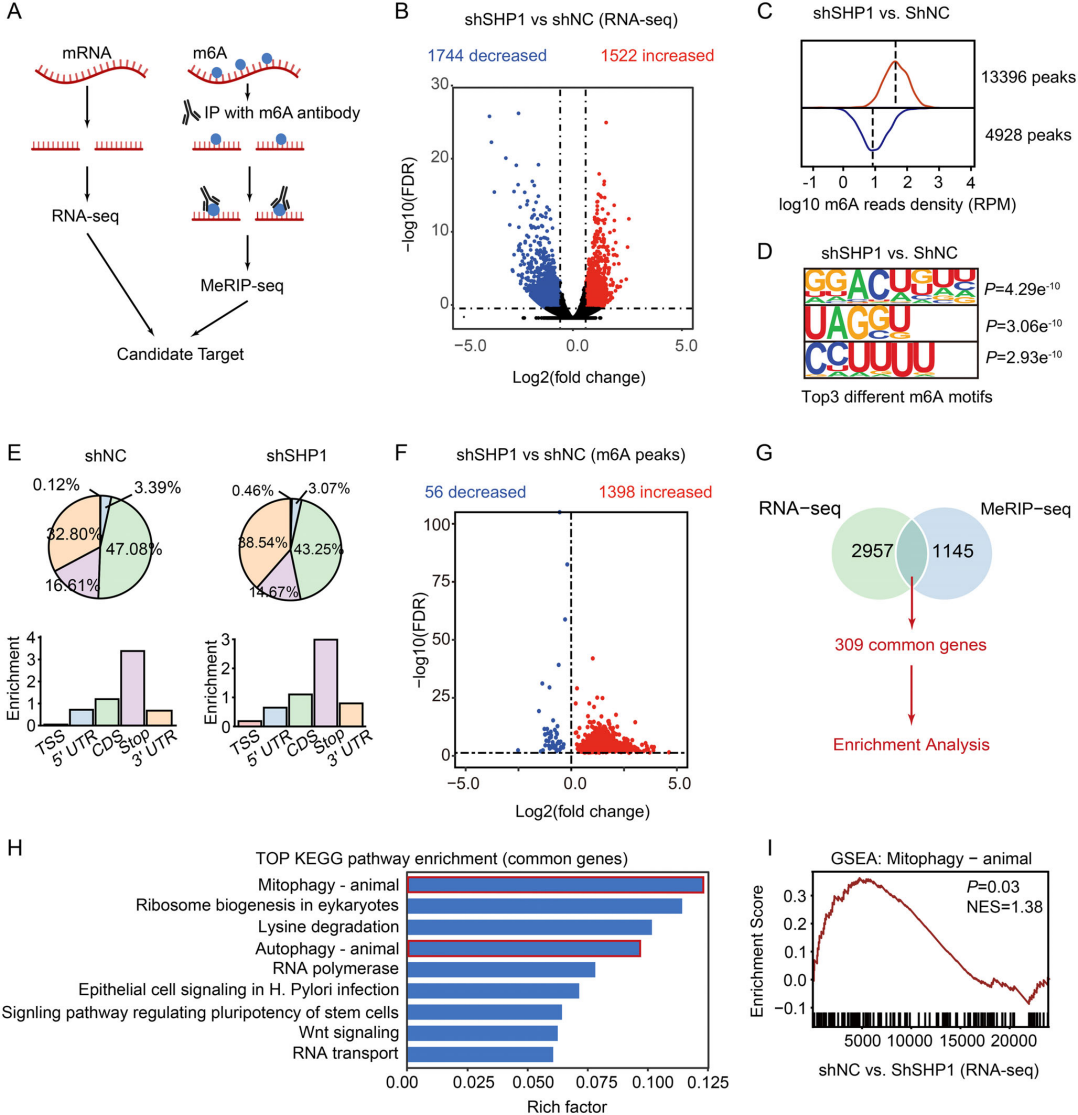
Integrated RNA‐seq and MeRIP‐seq reveal mitophagy as a key target of the SHP1/FTO axis in CRC. (A) Schematic representation of the combined RNA‐seq and MeRIP‐seq assays, used to screen targets of SHP1 in CRC. (B) Volcano plot depicting the differential gene expression between shSHP1 and shNC cells. (C) m6A modification profiles from MeRIP‐seq showing increased m6A peaks in shSHP1 cells. (D) Motif analysis of m6A‐enriched regions revealed that the GGACU motif is the top‐ranked increased motif in shSHP1 cells. (E) Distribution of m6A‐modified sites across the transcriptomes of shSHP1 and shNC cells. (F) Volcano plot showing differential m6A peaks between shSHP1 and shNC cells. (G) Venn diagram showing the overlap between differentially expressed genes from RNA‐seq and m6A‐modified genes from MeRIP‐seq. (H) Functional enrichment analysis of the 309 common genes. (I) Gene Set Enrichment Analysis showing significant alteration of the mitophagy pathway based on RNA‐seq data. Abbreviations: MeRIP‐seq, methylated RNA immunoprecipitation sequencing; FDR, false discovery rate; RPM, reads per million; TSS, transcription start site; UTR, untranslated region; CDS, coding sequence; NES, normalized enrichment score.

### 2.6 The SHP1–FTO Axis Regulated PTEN‐Induced Kinase 1 Expression in an m6A‐Dependent Manner

Mitophagy has been reported to be involved in the progression and malignancy of various cancers. This process involves two major pathways, namely, a ubiquitin‐dependent pathway mediated by PTEN‐induced kinase 1 (PINK1) and E3 ubiquitin–protein ligase parkin (Parkin) and a ubiquitin‐independent pathway involving BCL2 interacting protein 3 (BNIP3) and BCL2 interacting protein 3 like (NIX, also known as BNIP3L) [18]. By analyzing the common genes from the RNA‐seq and MeRIP‐seq results, we found that *PINK1*, rather than genes from the BNIP3–NIX axis, showed significantly decreased mRNA expression but increased m6A enrichment following SHP1 knockdown (Figure 5A–C). Consistently, a positive correlation between SHP1 and PINK1 mRNA expression was observed in TCGA‐COAD (Figure 5D), whereas a minimal correlation was found for genes in the BNIP3–NIX axis (Figure S7E,F). Furthermore, quantitative PCR (qPCR) analysis showed decreased *PINK1* expression following SHP1 knockdown, whereas the overexpression of SHP1, but not SHP1–C453S, increased PINK1 mRNA expression (Figure 5E). Moreover, treatment with TPI‐1 decreased *PINK1* expression, whereas treatment with the agonist SC43 had the opposite effect (Figure 5F). Meanwhile, the overexpression of FTO abrogated the change in *PINK1* mRNA after SHP1 knockdown, whereas overexpression of the unphosphorylated mutant FTO–Y475F did not have a similar effect, indicating FTO–m6A‐dependent *PINK1* degradation (Figure 5G). Consistently, the RNA stability assay showed the delayed elimination of *PINK1* mRNA in shNC cells compared with that in shSHP1 cells (Figure 5H). These results were validated at the protein level through immunoblotting (Figure 5I–L). Taken together, these findings collectively identify PINK1 as a key target of the SHP1–FTO–m6A axis.

**FIGURE 5:**
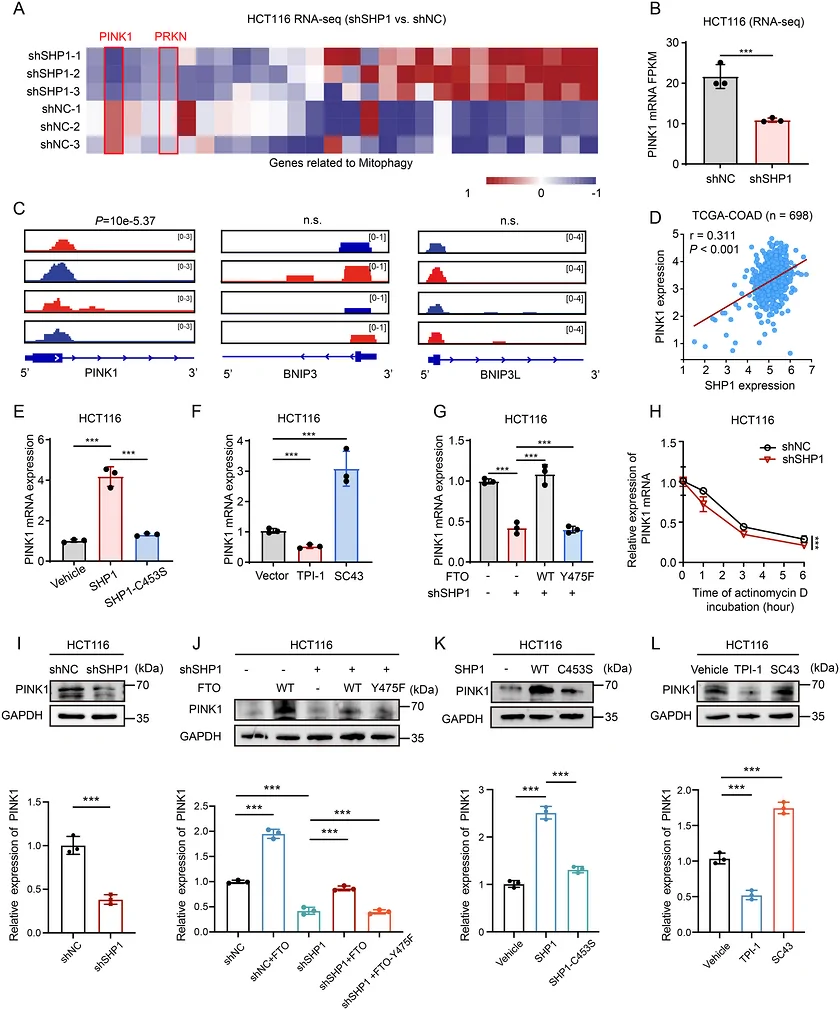
SHP1 regulates PINK1 expression in an m6A‐dependent manner. (A) Heatmap showing differentially expressed genes between shSHP1 and shNC cells, with a marked decrease in PINK1 expression upon SHP1 knockdown. (B) RNA‐seq showing decreased *PINK1* expression upon SHP1 knockdown in HCT116 cells. (C) Normalized m6A peak levels are shown in the shNC and shSHP1 groups. (D) Correlation analysis of *SHP1* and *PINK1* expression based on TCGA‐COAD data. (E) qPCR analysis indicating that the overexpression of SHP1 increased *PINK1* expression, whereas the SHP1–C453S mutant failed to restore *PINK1* levels. (F) Treatment with TPI‐1 led to decreased *PINK1* mRNA expression, whereas SC43 increased *PINK1* mRNA expression in HCT116 cells. (G) The overexpression of FTO in shSHP1 cells reversed the reduction in *PINK1* expression, whereas the FTO–Y475F mutant failed to restore *PINK1* levels. (H) actinomycin D RNA stability assay showing delayed degradation of *PINK1* mRNA in control cells compared with that in SHP1‐depleted cells, suggesting that SHP1 influences *PINK1* mRNA stability. (I) Immunoblotting to detect PINK1 protein levels in SHP1‐depleted cells. (J) HCT116 cells with SHP1 depletion were treated with FTO or the FTO–Y475F mutant, and western blotting was performed to determine PINK1 protein levels. (K) Western blotting results indicated that the overexpression of SHP1 increased PINK1 expression, whereas the SHP1–C453S mutant failed to restore PINK1 levels. (L) Immunoblotting results indicated that TPI‐1 led to decreased PINK1 protein expression, whereas SC43 increased PINK1 protein expression. *Abbrevaitions*: PRKN, parkin RBR E3 ubiquitin protein ligase; FPKM, fragments per kilobase of transcript per million mapped reads; n.s., not significant.

### 2.7 Heterogeneous Nuclear Ribonucleoprotein C Was Identified as the m6A Reader in SHP1–FTO–m6A‐Mediated PINK1 Regulation

To further identify the m6A reader protein involved in the regulation of SHP1/FTO/PINK1 axis, we performed an m6A RNA pulldown assay using the PINK1 m6A‐modified RNA probe, followed by liquid chromatography–tandem mass spectrometry (LC–MS/MS) analysis (Figure 6A). Among the enriched RNA‐binding proteins, heterogeneous nuclear ribonucleoprotein C (HNRNPC) was identified as a potential m6A reader candidate associated with PINK1 m6A RNA (Figure 6B). Subsequent immunoblotting of the RNA pulldown products confirmed that both FTO and HNRNPC were enriched in the PINK1 m6A–RNA‐bound complex, supporting their physical association with PINK1 m6A‐modified RNA (Figure 6C). Consistently, analysis of the TCGA‐COAD dataset revealed a significant negative correlation between HNRNPC and PINK1 expression, further supporting a potential regulatory relationship between these two molecules in CRC (Figure 6D). Functionally, knockdown of HNRNPC significantly increased both PINK1 mRNA expression (Figure 6E) and PINK1 protein abundance (Figure 6F), indicating that HNRNPC contributes to m6A‐mediated destabilization of PINK1 RNA. Furthermore, HNRNPC depletion reversed the reduction in PINK1 mRNA expression induced by SHP1 knockdown (Figure 6G), suggesting that HNRNPC acts downstream of the SHP1/FTO axis in regulating PINK1 RNA stability. Collectively, these results demonstrate that the SHP1/FTO axis modulates PINK1 expression by regulating HNRNPC recognition of m6A‐modified PINK1 RNA.

**FIGURE 6:**
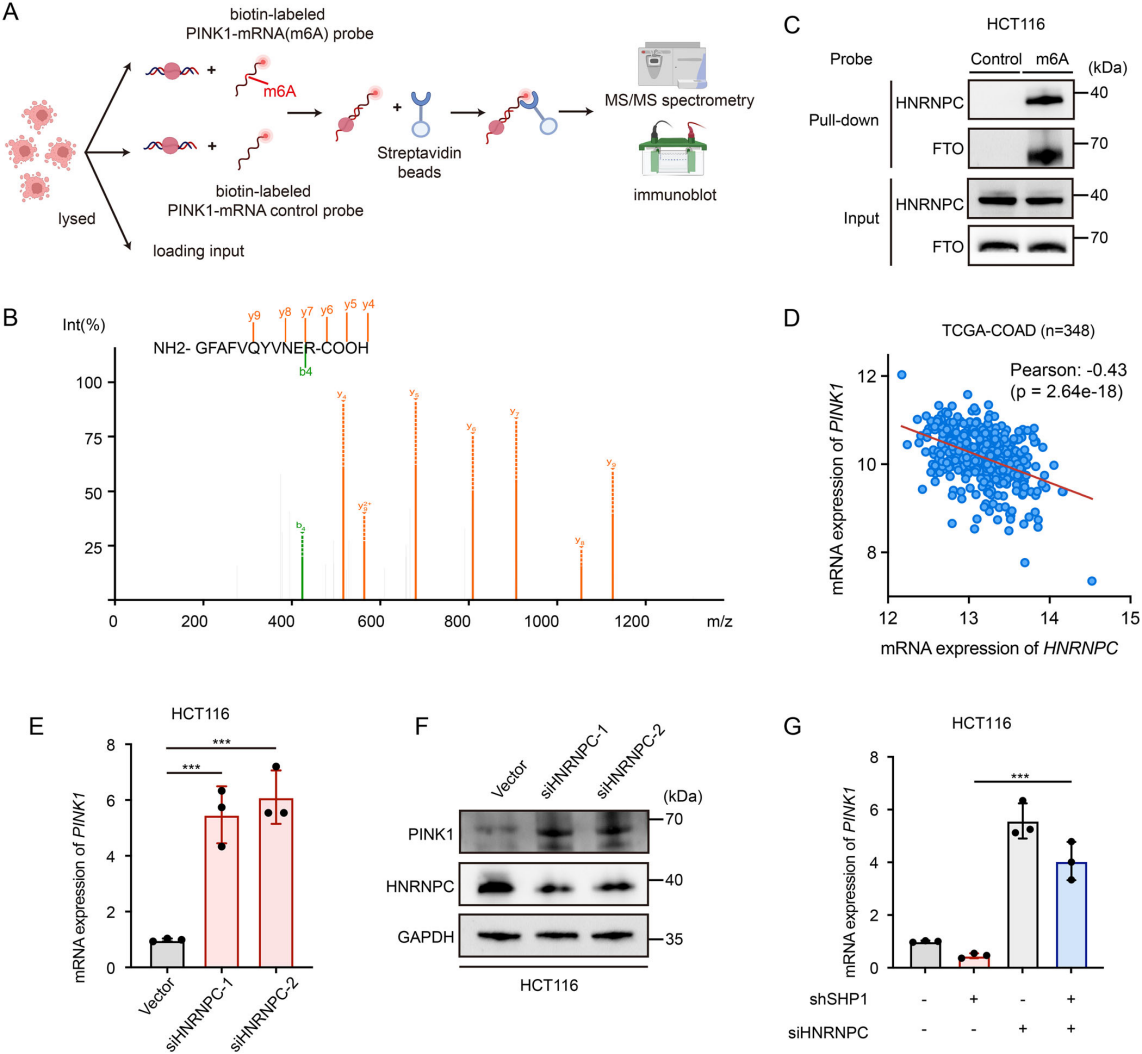
HNRNPC mediates m6A‐dependent regulation of PINK1 expression downstream of the SHP1/FTO axis. (A) Schematic illustration of the m6A RNA pull‐down assay. Biotin‐labeled PINK1 mRNA probes containing the m6A‐modified site or control probes were incubated with HCT116 cell lysates, followed by streptavidin bead capture and subsequent LC–MS/MS or immunoblot analysis. (B) Representative MS/MS spectrum identifying HNRNPC as a candidate protein enriched in the PINK1–m6A RNA pull‐down complex. (C) Immunoblot analysis of RNA pull‐down products showed that both HNRNPC and FTO were enriched by the PINK1–m6A RNA probe compared with the control probe, indicating their association with m6A‐modified PINK1 RNA. Input lysates were used as loading controls. (D) Correlation analysis of the TCGA‐COAD cohort showed a significant negative correlation between HNRNPC and PINK1 mRNA expression levels. (E) RT‐qPCR analysis showed that knockdown of HNRNPC markedly increased PINK1 mRNA expression in HCT116 cells. (F) Immunoblot analysis confirmed that HNRNPC knockdown increased PINK1 protein abundance. (G) RT‐qPCR analysis showed that HNRNPC knockdown reversed the reduction of PINK1 mRNA expression induced by SHP1 knockdown. Data are presented as mean ± SEM. Abbreviations: RT‐qPCR, reverse transcription quantitative polymerase chain reaction; SEM, standard error of the mean, ***p < 0.001.

### 2.8 The SHP1–FTO Axis Regulates Mitophagy in CRC

Next, we validated the functional role of SHP1 in the regulation of mitophagy. As shown in Figure 7A–C, transmission electron microscopy (TEM) revealed that mitochondrial autophagy was significantly reduced in HCT116 and HT29 cells, as well as in the corresponding xenograft tumors following SHP1 knockdown (Figure S8A,B). Additionally, a qPCR analysis showed a significant increase in the mitochondrial DNA (mtDNA) content after SHP1 depletion (Figure 7D,E). Consistently, JC‐1 staining demonstrated a significant reduction in the colocalization of JC‐1 monomers and JC‐1 aggregates in SHP1‐depleted cells (Figure 7F). Flow cytometry analysis further revealed that SHP1 knockdown restored the mitochondrial membrane potential (MMP), which was abrogated by the mitophagy agonist carbonyl cyanide m‐chlorophenylhydrazone (CCCP) (Figure 7G,H). Immunofluorescence assay also showed restored mitochondrial length in shSHP1 cells (Figure S8C,D). These data indicate that SHP1 knockdown disrupts mitophagy‐associated mitochondrial homeostasis. Consistent with the disruption of mitophagy and mitochondrial homeostasis, SHP1 knockdown significantly increased relative ATP content in both HT29 and HCT116 cells (Figure S8E), suggesting that SHP1 depletion alters the mitochondrial bioenergetic state of CRC cells. To further quantitatively assess mitophagy flux, we performed mt‐Keima assays. As shown in Figures 7I,J and S9A, SHP1 depletion markedly reduced the acidic mt‐Keima signal and decreased the 561/458 nm fluorescence ratio, indicating impaired delivery of mitochondria to lysosomes. Consistently, colocalization analyses of mitochondria with lysosomal and autophagosomal markers revealed that SHP1 depletion markedly reduced mitochondria–lysosome colocalization and colocalization of mitochondria with microtubule‐associated protein 1 light chain 3 (LC3) (Figure 7K–M), highlighting impaired mitophagy execution after SHP1 downregulation. Furthermore, immunoblot analysis showed that SHP1 knockdown led to decreased expression of LC3II, whereas levels of sequestosome 1 (SQSTM1/p62) and the PINK1‐downstream proteins translocase of outer mitochondrial membrane 20 (TOMM20) and translocase of inner mitochondrial membrane 23 (TIMM23) were increased (Figures 7N and S8F). Similarly, TPI‐1 treatment resulted in the concentration‐dependent modulation of these protein levels, indicating an on‐target effect of SHP1 in controlling mitophagy (Figures 7O and S8G). Collectively, these findings confirm that SHP1 depletion inhibits mitophagy signaling.

**FIGURE 7:**
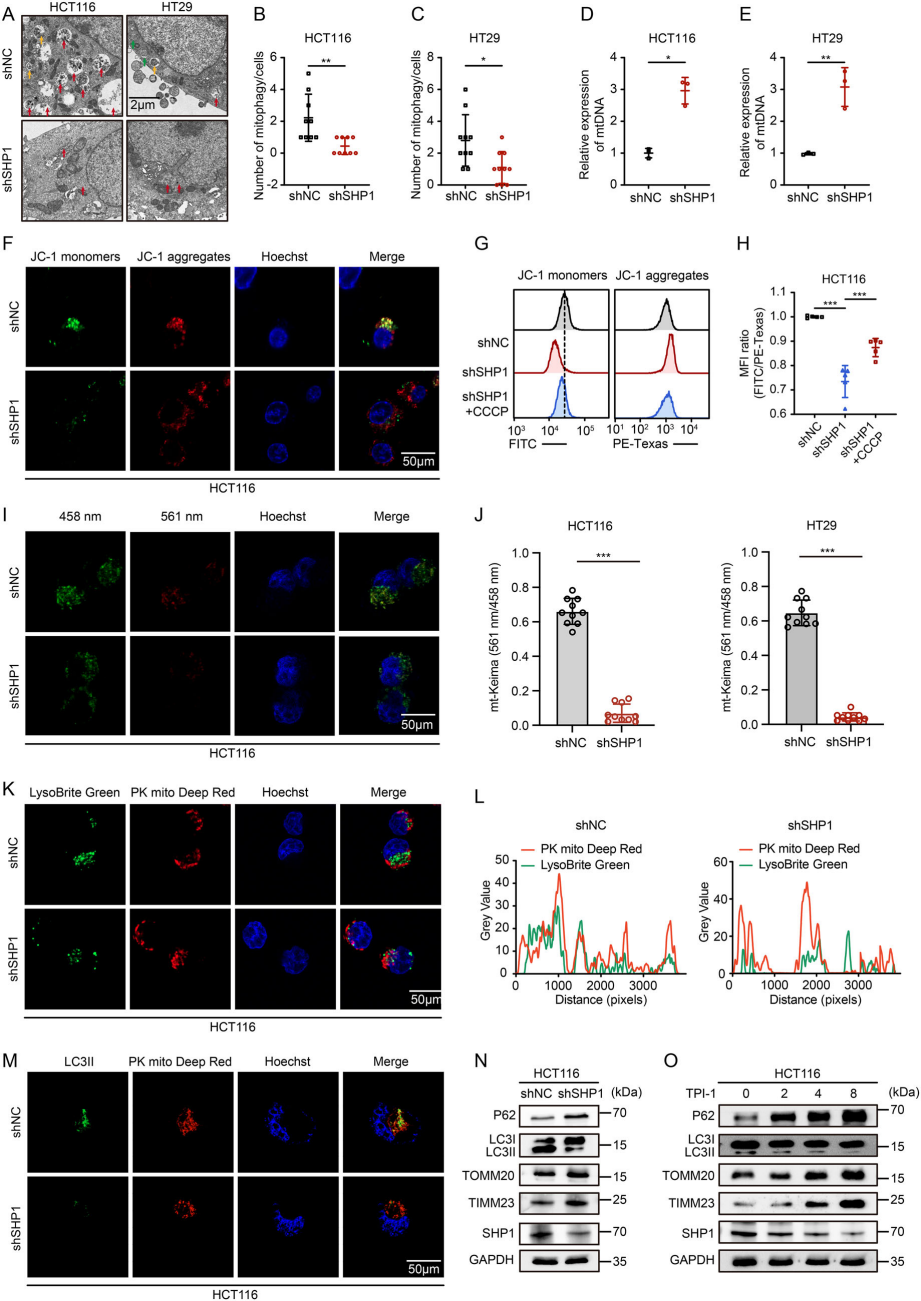
SHP1 depletion suppresses mitophagy and alters mitochondrial homeostasis in CRC cells. (A) Representative transmission electron microscopy images of HCT116 and HT29 cells with or without SHP1 knockdown. Red arrowheads indicate autolysosomes, green arrowheads indicate autophagosomes, and yellow arrowheads indicate mitophagosomes. (B and C) Quantification of mitophagy‐related structures based on TEM images in HCT116 (B) and HT29 (C) cells. (D and E) Relative mtDNA content measured by qPCR in HCT116 (D) and HT29 (E) cells after SHP1 knockdown. (F) Representative JC‐1 staining images showing mitochondrial membrane potential in HCT116 cells with or without SHP1 knockdown. (G) Flow cytometric analysis of JC‐1 fluorescence in shNC, shSHP1, and shSHP1 cells treated with CCCP. (H) Quantification of the JC‐1 fluorescence ratio based on flow cytometry. (I) Representative mt‐Keima images showing mitophagy flux in HCT116 cells with or without SHP1 knockdown. (J) Quantification of the mt‐Keima 561/458 nm fluorescence ratio in HCT116 and HT29 cells. (K) Representative immunofluorescence images showing lysosome–mitochondria colocalization in HCT116 cells. LysoBrite Green was used to label lysosomes, PK Mito Deep Red was used to label mitochondria, and Hoechst was used for nuclear counterstaining. (L) Representative line‐scan intensity profiles showing lysosome–mitochondria colocalization. (M) Representative immunofluorescence images showing LC3–mitochondria colocalization in HCT116 cells. (N) Immunoblot analysis of mitophagy‐related proteins in HCT116 cells with or without SHP1 knockdown. (O) Immunoblot analysis of mitophagy‐related proteins in HCT116 cells treated with increasing concentrations of TPI‐1. Abbreviations: MFI, mean fluorescence intensity; mt‐Keima, mitochondria‐targeted Keima fluorescent reporter. *p < 0.05, **p < 0.01, ***p < 0.001.

To further determine the functional relevance of the FTO Y475 residue in regulating tumor cell growth and mitophagy, we performed functional assays in cells overexpressing the FTO–Y475F mutant. As shown in Figure S10, compared with wild‐type FTO, FTO–Y475F failed to fully promote cell proliferation in CCK‐8 assays and showed a markedly reduced capacity to support colony formation in both HCT116 and HT29 cells. Similarly, mt‐Keima assays showed that wild‐type FTO promoted mitophagy flux, whereas FTO–Y475F failed to fully support this effect (Figure S9B,C). Consistently, JC‐1 staining showed that cells expressing FTO–Y475F displayed impaired MMP compared with those expressing wild‐type FTO (Figure S11A). Moreover, lysosome–mitochondria colocalization was markedly reduced in FTO–Y475F‐expressing cells (Figure S11B,C). Together, these findings indicate that the Y475 residue is required for SHP1/FTO‐mediated cancer cell growth as well as mitophagy flux and mitochondrial homeostasis in CRC cells.

### 2.9 SHP1 Depletion Showed Antitumor Efficacy in CRC

Given the significant role of SHP1 in CRC malignancy, we investigated the translational potential of SHP1 depletion. First, we used TPI‐1, a well‐established SHP1 inhibitor, at concentrations within the previously reported SHP1‐selective range [16]. As shown in Figure 8A–C, TPI‐1 treatment significantly delayed the growth of HCT116 and HT29 xenograft tumors. Furthermore, TEM analysis of tumor samples revealed that SHP1 inhibition significantly suppressed mitophagy in both HCT116 and HT29 tumors, which is consistent with our previous findings (Figure 8D). Next, we specifically developed a vesicle for the targeted delivery of SHP1–siRNA by using lipid nanoparticles (LNPs) (Figure 8E,F). The LNP–siRNAs were visualized by TEM (Figure S12A) and showed a normal hydrodynamic diameter distribution (Figure S12B). LNP treatment showed no obvious systemic toxicity in mice (Figure S12C). The in vivo knockdown efficiency of LNP–siSHP1 was confirmed by western blot assays using proteins harvested from xenograft tumors (Figure S12D). Compared with that in mice receiving LNP–siNC treatment, treatment with LNP–siSHP1‐1 and LNP–siSHP1‐2 resulted in the significant suppression of HCT116 and HT29 tumor growth (Figure 8G–I). Collectively, these results suggest that SHP1 depletion, using either an inhibitor or siRNA–LNP delivery, significantly attenuates CRC tumor progression. Our findings suggest that SHP1 might be a promising therapeutic target for CRC treatment.

**FIGURE 8:**
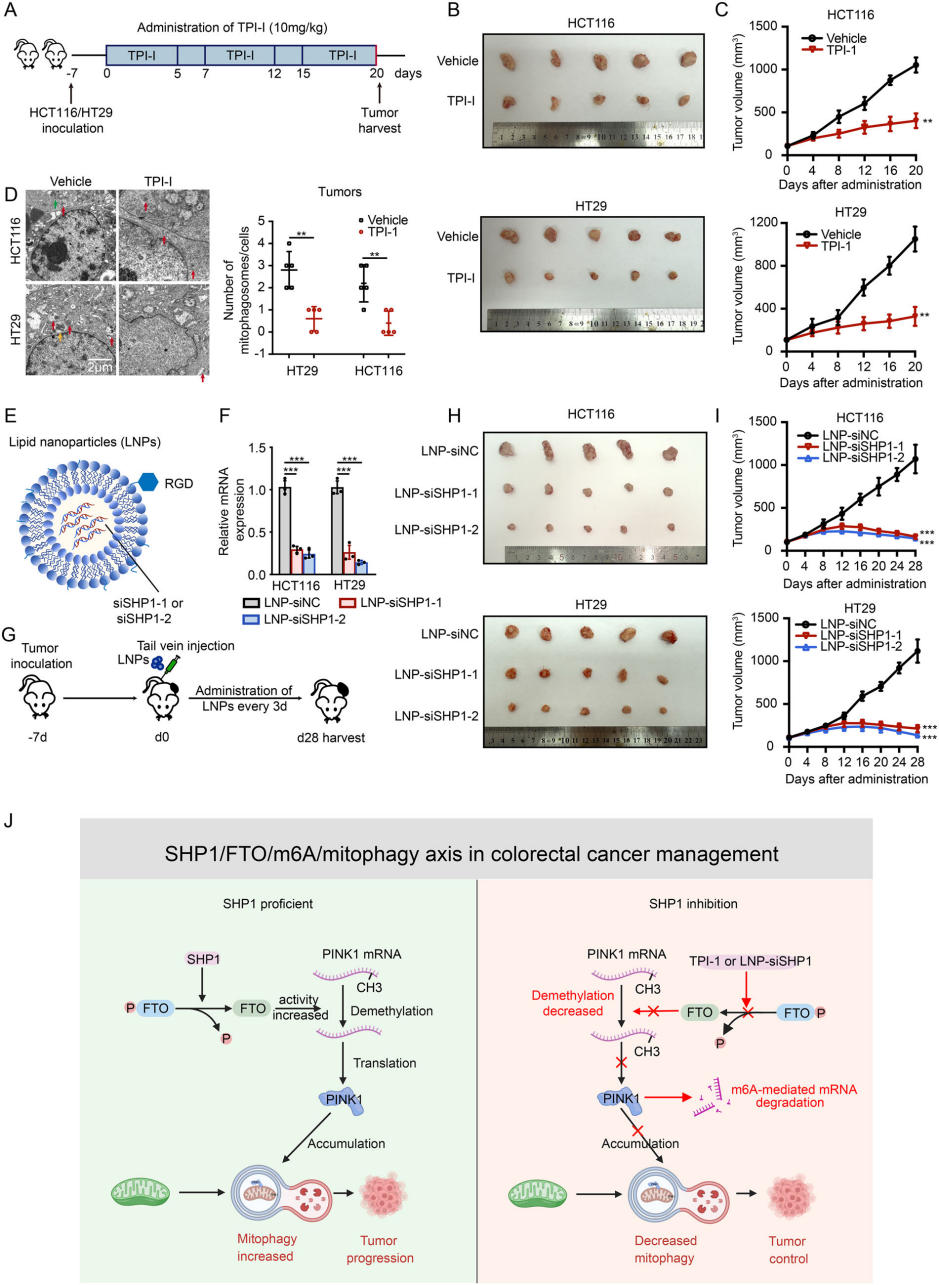
SHP1 depletion suppresses tumor growth and mitophagy in CRC. (A) Flowchart depicting the administration of TPI in animal models. (B) Representative images of HCT116 and HT29 xenograft tumors from mice treated with TPI‐1. (C) Tumor growth curves from nude mice. (D) TEM analysis of HCT116 and HT29 xenograft tumor samples, showing reduced mitophagy in TPI‐1‐treated tumors (red arrowheads: autolysosome; green arrowheads: autophagosome; yellow arrowheads: mitophagosome). (E) Schematic depiction of LNP–siRNA delivery system for targeted SHP1 knockdown in CRC tumors. (F) qPCR analysis was applied to validate the efficiency of LNP–siRNA. (G) Flowchart depicting the administration of LNP–siRNA to animal models. (H and I) Representative images and tumor volume curves showing delayed growth in mice treated with LNP–siSHP1 compared with that in controls. (J) Proposed model of the SHP1/FTO/m6A/PINK1 axis in CRC. Under SHP1‐proficient conditions, SHP1 dephosphorylates FTO at Y475, enhances FTO demethylase activity, reduces m6A modification on PINK1 mRNA, maintains PINK1 expression, and promotes PINK1‐dependent mitophagy and tumor progression. Conversely, SHP1 inhibition increases FTO phosphorylation, decreases FTO demethylase activity, enhances m6A‐dependent degradation of PINK1 mRNA, suppresses PINK1‐mediated mitophagy, and ultimately inhibits CRC tumor growth. Abbreviation: RGD, arginine–glycine–aspartic acid.

## 3 Discussion

CRC is characterized by its complex biological features, underscoring the urgent need for the development of novel biomarkers that can not only predict prognosis more accurately but also serve as therapeutic targets. In this study, we elucidated a novel role for SHP1 in CRC progression, particularly through its regulatory effects on FTO and subsequent modulation of m6A RNA methylation levels. Our results demonstrate that SHP1 can dephosphorylate FTO at the Y475 site, in turn enhancing the demethylase activity of FTO. This alteration leads to global changes in the m6A methylation patterns, which subsequently affect downstream gene PINK1 expression and PINK1‐mediated mitophagy. We further showed that pharmacological inhibition or siRNA‐mediated depletion of SHP1 suppresses CRC tumor growth in xenograft models, providing a mechanistic rationale for future investigation of SHP1‐targeted intervention in CRC.

As a member of the tyrosine phosphatase family, SHP1 functions primarily by dephosphorylating key mediators of important biological processes [6]. Previous studies have highlighted its importance in various immune responses, including T cell‐mediated antitumor effects, NK cell‐mediated immune killing, and macrophage‐mediated phagocytosis [7, 19, 20, 21]. Additionally, SHP1 has been implicated in the development and progression of hematological malignancies, such as lymphocytic and granulocytic leukemia, and accumulating evidence suggests that its depletion can effectively inhibit the growth of hematopoietic tumors [22, 23, 24, 25]. However, the role of SHP1 in solid tumors was less explored. Prior research on tumoral SHP1 has been largely focused on its classical substrate, STAT3 [25, 26, 27]. By directly dephosphorylating STAT3, SHP1 regulated its downstream signaling pathways and thus modulates key oncogenic processes, such as tumor cell proliferation, apoptosis, epithelial–mesenchymal transition, metastasis, and chemo‐resistance [25, 26, 27]. Our previous study demonstrated that SHP1 acts as a signaling hub for antitumor immune responses in response to the combination of radiotherapy and immunotherapy in CRC [10]. This observation motivated us to perform a more comprehensive analysis of its role in the context of CRC. In this study, we found that SHP1 is highly expressed in CRC, and this elevated expression was found to correlate with poor clinical outcomes. Functional studies revealed that SHP1 promotes the malignant behaviors of CRC, including enhanced tumor growth, proliferation, migration, and invasion, underscoring its oncogenic potential in CRC. A further enrichment analysis of SHP1‐interacting proteins revealed a new function for SHP1 in RNA metabolic processes, particularly mRNA metabolism and degradation. Given that RNA methylation is one of the most common modifications influencing RNA degradation, we were able to identify a novel substrate of SHP1, FTO, a well‐recognized m6A demethylase. We further demonstrated that SHP1 interacts with FTO in CRC cells. This interaction appeared to affect FTO function, potentially altering RNA metabolic processes, including RNA stability and translation. Dysregulated RNA metabolism is becoming increasingly recognized as a hallmark of cancer [28]. Our findings suggested a previously uncharacterized axis involving SHP1 and FTO in RNA metabolism, which adds a new layer to our understanding of the SHP1 functional network in cancer.

FTO was the first identified demethylase of RNA m6A [14]. Since the interaction between SHP1 and FTO relies on SHP1 phosphatase activity, we speculated that SHP1 could modulate FTO dephosphorylation. Further results verified that SHP1 directly dephosphorylates FTO at the tyrosine residue Y475. FTO has been reported to undergo multiple posttranslational modifications, including phosphorylation, SUMOylation, ubiquitination, and acetylation. Tomizawa et al. demonstrated that the phosphorylation of FTO–Thr150 controls its nuclear shuttling and thus influences the mRNA expression of cell cycle‐related proteins [17]. Similarly, the SUMOylation of SHP1 can regulate FTO demethylase activity, thereby promoting the methylation of oxidative stress‐related genes [29]. Moreover, through mutational analyses of potential FTO phosphorylation sites and crystallization of the FTO–RNA complex, an intensive study demonstrated that the phosphorylation at specific residues could also directly alter the affinity of FTO for its RNA substrates [30]. Based on these findings, we speculated that the dephosphorylation of FTO, mediated by SHP1, modulates its demethylase activity. The introduction of FTO–Y475F significantly increased global m6A levels, indicating the direct effect of Y475 phosphorylation on FTO activity. Collectively, our findings revealed a novel posttranslational regulatory mechanism of FTO. Given the critical roles of m6A in RNA metabolism and tumor biology, this mechanism provides new insights into the regulation of FTO activity and its contribution to CRC progression.

Previous studies have demonstrated that FTO is highly expressed in various solid tumors and regulated multiple biological processes, including cell cycle progression, apoptosis, oxidative stress, and cancer stemness maintenance [14, 31]. In CRC, FTO is predominantly characterized as a potent oncogenic driver that stabilizes critical prosurvival and progrowth transcripts, such as snail family transcriptional repressor 1 and MYC proto‐oncogene basic helix‐loop‐helix transcription factor (MYC), through its m6A demethylase activity, thereby promoting epithelial–mesenchymal transition, and tumor proliferation [12]. Furthermore, comprehensive analyses of multiple CRC databases demonstrate that elevated FTO expression is significantly associated with poorer clinical outcomes, particularly in primary tumors and those resistant to 5‐FU chemotherapy [32]. These findings are consistent with our results showing that higher levels of SHP1 lead to enhanced FTO activity and, consequently, poorer survival for patients with CRC. However, FTO is also reported to act as a tumor suppressor under certain conditions—such as during hypoxia, where its downregulation facilitates metastasis by modulating metastasis‐associated protein 1 m6A levels [33]. In our study, FTO's demethylase activity is specifically toggled by SHP1‐mediated dephosphorylation at Y475, a posttranslational modification that allows FTO to exert its oncogenic effects, such as the stabilization of PINK1 to drive mitophagy‐mediated survival, regardless of its total mRNA abundance. These results highlight that m6A modification repertoire is selectively different under different physiological conditions, dictated not only by FTO expression but by the precise tuning of its enzymatic activity by upstream regulators like SHP1.

To further map the m6A atlas affected by the SHP1–FTO regulatory axis and the underlying mechanisms in driving CRC progression, we conducted integrated meRIP‐seq and RNA‐seq analyses. This comprehensive approach allowed for the identification of common genes exhibiting differential mRNA expression and m6A modifications following SHP1 knockdown. Functional enrichment results highlighted “mitophagy” as the most significantly altered pathway among the common genes, with PINK1 being the key gene altered in the SHP1/FTO regulation. We further provided a multidimensional characterization of how the SHP1/FTO/PINK1 axis governs mitophagy dynamics. Specifically, SHP1‐mediated dephosphorylation of FTO at Y475 enhances its demethylase activity to stabilize PINK1 mRNA, thereby preventing its degradation and ensuring robust PINK1‐mediated mitophagy to maintain mitochondrial quality and cellular energy. As a vital process required for maintaining mitochondrial homeostasis, mitophagy can act as either an inhibitor or promoter of tumor malignancy depending on the tumor microenvironment [18, 34]. Nevertheless, the inhibition of mitophagy or autophagosome–lysosome fusion has strong antitumor effects on CRCs [35, 36], which aligns with the findings of our study. Interestingly, FTO was also reported to promote mitophagy by preventing the m6A‐dependent degradation of BNIP3 mRNA during sepsis progression [37], which is different from our results showing that the focus of SHP1‐mediated FTO functional enhancement occurs specifically through regulation of the PINK1–Parkin pathway. These findings underscore the context‐dependent nature of m6A modifications and their dynamic roles under different physiological and pathological conditions. Collectively, these data suggest that SHP1‐mediated FTO dephosphorylation could inhibit m6A‐dependent PINK1 mRNA degradation, which in turn promotes PINK1‐mediated mitophagy. This newly discovered SHP1–FTO pathway may function as a therapeutic target for controlling CRC development.

Recently, improvements in phosphatase inhibitors have been relatively robust. For example, Src homology 2 domain‐containing PTP 2 (SHP2) inhibitors have shown promising antitumor efficacy in combination therapy and against CRC with hyperactive rat sarcoma signaling [38]. Multiple SHP2 inhibitors, such as RMC‐4630 and TNO155, have been tested in clinical trials, while inhibitors of another phosphatase, protein phosphatase 2A, have also shown promising efficacy in cancer therapy by dephosphorylating oncogenic substrates like MYC and protein kinase B [39, 40]. TPI‐1 is a potent SHP1 inhibitor that has exhibited antitumor activity in melanoma models through T‐cell‐dependent mechanisms associated with increased IFN‐γ production [16]. Although TPI‐1 can inhibit both SHP1 and SHP2, its inhibitory activity is concentration dependent, with biochemical assays demonstrating approximately 10‐fold greater potency against SHP1 than SHP2. Importantly, the concentrations used in the present study were within the previously reported SHP1‐selective range, in which TPI‐1 increased the phosphorylation of SHP1 substrates while having little effect on SHP2‐associated signaling [16]. Together with the concordant effects of genetic SHP1 depletion and evidences from the TCGA‐database, these findings support SHP1 inhibition as the principal mechanism underlying the effects of TPI‐1 observed in our CRC models. However, previous literature has identified the SHP1 agonist SC43 as a potential treatment for CRC by antagonizing the STAT3 pathway. This apparent discrepancy likely reflects a “delicate balance” of SHP1 activity, where its functional role—acting either as a tumor suppressor or an oncogene—is highly context dependent and determined by the dominant downstream substrates in a given microenvironment. In our model, the prosurvival benefits derived from SHP1‐mediated mitochondrial quality control appear to outweigh its canonical inhibitory effects on STAT3 signaling. Furthermore, SHP1 inhibition, whether through direct inhibition by TPI‐1, or by indirect regimens such as ATRi and Vitamin E, exerted potent immune‐activating effect and offers superior translational potential when combined with immunotherapy [10, 41, 42]. In the current study, we showed that targeting SHP1 with siRNAs and TPI‐1 could inhibit CRC development in an immunodeficient model, primarily by regulating PINK1‐mediated mitophagy, highlighting a new mechanism through which tumoral SHP1 promotes tumor growth. Collectively, these results indicate the vast clinical potential for targeting SHP1.

Our study has high clinical relevance. First, we stratified the prognostic value of SHP1 for CRC management. Second, we provide a molecular basis for targeting SHP1, either by using TPI‐1 or through the delivery of siRNAs via LNPs, in clinical investigations on CRC. FTO inhibition and mitophagy were reported to correlate with chemoresistance in CRC [12, 13, 32, 43]. By perturbation of mitochondrial homeostasis, SHP1 inhibition could thus be tested in combination with chemotherapy or used to reverse chemoresistance, making it a promising direction for cancer management.

Our study had some limitations. First, CRC cell lines and xenograft tumors do not represent the natural context of human CRCs owing to the high heterogeneity and microenvironment differences. Therefore, models, such as organoids derived from human CRCs and primary tumor models in mice, are required to further clarify the therapeutic effect of SHP1 depletion on CRC. Second, despite the on‐target effect of TPI‐1 observed in the current study, more potent SHP1 inhibitors should be developed because the inhibition of SHP2 and other phosphatases with similar structures has been reported with high doses of TPI‐1 [9]. Third, we focused on SHP1‐mediated FTO dephosphorylation and subsequent RNA modification changes. Future studies are required to evaluate the phosphoproteomic landscape after SHP1 inhibition; these may contribute to a more thorough understanding of SHP1 functions in tumor cells.

In conclusion, this study shows that SHP1‐mediated FTO dephosphorylation regulates mitophagy in an m6A‐dependent manner (Figure 8J). The SHP1–FTO–m6A network plays an essential role in CRC progression and may represent a candidate therapeutic vulnerability in CRC.

## 4 Materials and Methods

### 4.1 Cell Culture

The CRC cell lines HCT116 and HT29 were sourced from the Cell Bank of the Chinese Academy of Sciences (Shanghai, China). HCT116 and HT29 cells were cultured in McCoy's 5A modified medium (HyClone, ThermoFisher, USA) supplemented with 10% fetal bovine serum (FBS; Gibco, ThermoFisher). Caco2 cells were grown in Dulbecco's modified Eagle's medium‐high glucose (HyClone, ThermoFisher), also supplemented with 10% FBS. These cell lines were all maintained in a controlled environment with 5% CO_2_, at a temperature of 37°C.

### 4.2 Reagents and Plasmids

The following compounds were employed in this investigation: SC43 (Medchemexpress; HY‐136657); TPI‐1 (Medchemexpress; HY‐100463); PK mito Deep Red (Nanjing Genvivo Biotech; PKMDR‐1); LysoBrite Green (AAT Bioquest; 22643); mt‐Keima (Hanbio Biotechnology; HH20251014WHZL‐ADP01); Hoechst 33342 (Beyotime Biotechnology; C1029); and MMP assay kit with JC‐1 (Beyotime Biotechnology; C2006). Plasmids expressing SHP1–WT, SHP1–C453S, and wild‐type FTO were obtained from Miaoling Biotechnology. Mutants of FTO plasmids including FTO–Y39A, FTO–Y108A, FTO–Y220A, FTO–Y475A, and FTO–Y475F were generated by PCR using Mut Express II Fast Mutagenesis Kit V2 (Vazyme; C214‐01) following the manufacturer's instructions. FTO–WT was used as the template.

### 4.3 Animals

All animal experiments were approved by the Laboratory Animal Welfare and Ethics Committee of Tongji Hospital, Huazhong University of Science and Technology (TJH‐201910007). For this, 5‐week‐old female Balb/c nude mice were maintained in a temperature‐controlled setting. A subcutaneous tumor model was created by implanting 5 × 10^5^ HT29 or HCT116 cells in 50 µL of sterile PBS subcutaneously into the right flanks of female Balb/c nude mice. The mice were randomly assigned to groups containing five tumors when the tumor size reached 100 mm^3^. Mice were then treated with either vehicle or TPI‐1 (MedChemExpress; HY‐100463) via intraperitoneal injection (10 mg/kg) twice per week. Vernier calipers were used to measure the tumor volume every 4 days. The tumor volume was calculated as *V* = (length × width^2^)/2. After treatment, the mice were euthanized, and the tumors were removed for analysis.

### 4.4 Cell Transfection

Cells were plated in six‐well plates 1 day prior to siRNA transfection. Lipofectamine 3000 Transfection Reagent (Invitrogen; L3000015) was used for siRNA transfection, following the manufacturer's guidelines. The cells were collected for further analysis between 48 and 72 h after transfection. The siRNA oligonucleotide sequences are listed in Table S2.

### 4.5 CCK‐8, EdU, and Colony Formation Assays

Cells were seeded into 96‐well plates at a density of 2000 cells/well in plating medium enriched with 10% FBS. Cell viability was assessed using the CCK‐8 protocol (CCK‐8; MedChemExpress). For the EdU incorporation assay, the cells were cultured in 12‐well plates at a density of 8 × 10^4^ cells/well. Postexposure to various experimental conditions, cells were incubated with a medium containing 50 µM EdU for 2 h. Subsequently, the cells were fixed in a 4% paraformaldehyde solution at room temperature for 20 min. The cells were then stained with Hoechst reagent and Apollo staining solution (RiboBio; C10310). A fluorescence microscope (Olympus IX71; Tokyo, Japan) was used to quantify the proportion of EdU‐positive cells in five randomly selected fields. Cells were seeded at a density of 600 cells/well in six‐well plates. After a 14‐day period, the number of colonies was counted and statistically analyzed.

### 4.6 Cell Migration and Invasion Assays

Wound healing and Matrigel invasion assays were conducted to evaluate the migratory and invasive capabilities of the cells. The cells were seeded at a density of 2 × 10^5^ cells/well in six‐well plates to initiate the migration process. Once the cells reached approximately 90% confluence, a 1 mL pipette tip was used to create a uniform wound by gently scraping the cell monolayer. The rate of wound closure was quantified using ImageJ software after a 24 h period. For the invasion experiments, a transwell chamber assay was employed. Matrigel (ABW Matrigengel 8,027,045) was applied to the upper chamber and incubated for 30 min at 37°C. Subsequently, cells were seeded in the top chamber in serum‐free medium at a density of 3 × 10^4^ cells per well, with 500 µL of medium containing 10% FBS added to the bottom chamber. A 4% paraformaldehyde solution was used to fix the cells that had penetrated the top membrane over a 24 h incubation period at 37°C in a 5% CO_2_ atmosphere. The cells were then stained with 0.1% crystal violet solution for 60 min. A microscope was used to enumerate the cells in five randomly selected fields.

### 4.7 Western Blot

Protein extraction was performed using RIPA lysis buffer procured from Beyotime and supplemented with a phosphatase inhibitor cocktail (HY‐K0021; MedChemExpress) and phenylmethylsulfonyl fluoride. Proteins were resolved on 10% sodium dodecyl sulfate‐polyacrylamide gel electrophoresis gels and transferred onto PVDF membranes. The membranes were initially blocked with bovine serum albumin for 1 h, followed by an overnight incubation at 4°C with the primary antibodies. Subsequently, the membranes were incubated for 1 h at ambient temperature with the corresponding secondary antibodies. The immunoblots were visualized using SuperSignal West Pico Chemiluminescent Substrate and the BOX Chemi X system (Syngene, Cambridge, UK). A comprehensive list of the specific primary and secondary antibodies used in this study is presented in Table S3.

### 4.8 Co‐IP

Cell lysates were prepared using NP40 lysis buffer (Beyotime), fortified with a phosphatase inhibitor cocktail and phenylmethylsulfonyl fluoride. Protein A/G magnetic beads (HY‐K0202; MedChemExpress) were pre‐incubated with either anti‐SHP1 (diluted 1:100), anti‐FTO (diluted 1:100), or anti‐Flag (diluted 1:100; Sigma, St. Louis, MO, USA) antibodies for 2 h at 4°C. Subsequently, following the manufacturer's protocol, the cell lysates were combined with the magnetic beads for an additional 4 h at 4°C. After extensive washing, bound proteins were eluted and analyzed by immunoblotting. Immunoblotting was subsequently employed to ascertain the presence of target proteins in the samples using the appropriate antibodies.

### 4.9 Real‐Time qPCR

Real‐Time qPCR was executed following a previously established protocol [44]. Briefly, total RNA was extracted from cellular samples using the TRIzol method. RNA was quantified using a NanoDrop 2000 spectrophotometer. Complementary DNA synthesis was performed using 5× PrimeScript RT Master Mix. For real‐time qPCR, the ChamQ SYBR qPCR Master Mix (Vazyme) was used on an ABI PCR system. Gene expression levels were determined relative to the expression of glyceraldehyde‐3‐phosphate dehydrogenase (GAPDH), which served as an endogenous control for normalization. The primer sequences used for qPCR are listed in Table S4.

### 4.10 Determination of the mtDNA Copy Number

A Steady Pure Universal Genomic DNA Extraction Kit (Accurate Biotechnology, China) was used to extract the genomic DNA. Next, TB Green Premix Ex Taq II (Takara) was added to the reaction, along with 100 ng of DNA and forward and reverse primers for mitochondrially encoded cytochrome *c* oxidase‐2 (*MT‐CO2*) (10 µM for each primer). The 2−ΔΔCt technique was used to calculate relative gene expression levels. *GAPDH*, an endogenous control gene, was used to determine the ΔCt value for each sample.

### 4.11 m6A Dot Blot Assay

Total RNA extracted from CRC cells was combined with incubation buffer and denatured for 5 min at 65°C to prepare it for the m6A dot blot. Following a 30 min UV crosslinking process, samples (800, 400, 200, or 100 ng) dissolved in 6× SSC buffer were spotted onto an Amersham Hybond‐N+ membrane (GE Healthcare, USA) that was placed on a Bio‐Dot Apparatus (Bio‐Rad, USA). After blocking for 1 h in PBST with 5% bovine serum albumin and 0.1% Tween 20, the membrane was incubated with an anti‐m6A antibody (1:2000; Abcam; #ab208577) overnight at 4°C. HRP‐conjugated anti‐rabbit IgG (1:5000) was then incubated with the sample for an additional hour, and the Clarity Western ECL Substrate (GE Healthcare; #1705061) was used for visualization. The entire input RNA was visualized on a membrane by staining it with 0.02% methylene blue.

### 4.12 Phos‐Tag Assay

SDS‐PAGE with Phos‐tag acrylamide gels (Wako chemicals) was utilized to identify phosphorylated proteins. Following electrophoresis, Phos‐tagged acrylamide gels were rinsed with a transfer solution containing 5 mM EDTA for 10 min with gentle shaking. Then, in accordance with the manufacturer's instructions, the gels were rinsed with transfer buffer containing no EDTA for 10 min. Using conventional immunoblotting techniques, proteins were transferred to PVDF membranes and identified using the corresponding antibodies.

### 4.13 RNA Stability Assay

Six‐well plates were inoculated with CRC cells exhibiting either the overexpression or knockdown of SHP1. At the initial time point, after a 24 h incubation, cells from a single well were harvested. To achieve a final concentration of 10 µg/mL, in 2 mL of culture medium, 20 µL of a 1 mg/mL actinomycin D (Sigma; catalog number A9415) stock solution was added to the remaining wells. Samples were collected 0, 1, 3, and 6 h after actinomycin D administration. RNA was extracted from cell pellets for subsequent qPCR analysis.

### 4.14 RNA Pull‐Down Assay

RNA pull‐down assay was carried out by using the RNA pulldown kit (BersinBio, China; #Bes5102). Briefly, biotin‐labeled PINK1–mRNA(m6A) and PINK1–mRNA(control) probes were incubated with cell lysates, followed by streptavidin magnetic beads incubation and subsequent washing under the manufacturer's instructions. RNA‐associated proteins were further eluted and subjected to LC–MS/MS analysis and SDS‐PAGE. Sequences of the probes were listed in the Table S5.

### 4.15 Flow Cytometry

MMP was assessed using a JC‐1 assay kit (Beyotime, Nantong, China). This assay involves a lipophilic dye that accumulates in intact mitochondria, resulting in the formation of red fluorescent J‐aggregates, whereas it forms green fluorescent J‐monomers in compromised mitochondria. Cells were stained with the JC‐1 probe and subsequently analyzed via flow cytometry to determine MMP levels.

### 4.16 Immunohistochemistry Staining and Scoring

CRC tissue arrays (HColA160Su02) were acquired from the Shanghai Outdo Biotech Company (Shanghai, China). This array encompasses a comprehensive sample cohort derived from 100 individuals with CRC. Immunohistochemistry (IHC) staining was performed in strict accordance with protocols described by Yeasen Biotechnology. To ensure objectivity, a quantitative assessment of protein expression across each specimen was conducted by a pathologist who was blinded to the clinical information. The modified H‐score metric, which includes the proportional distribution of weak (1+), moderate (2+), and intense (3+) staining, was employed to quantify protein expression levels.

### 4.17 MMP and Immunofluorescence Assays

Following the manufacturer's instructions, the cells were incubated with the fluorescent mitochondrial probe JC‐1 to assess the MMP (Beyotime). Cells were cultured using either LysoBrite Green (AAT Bioquest; 22643) or PK Mito Deep Red (Genvivo Biotech; PKMDR‐1) to mark the lysosomes and mitochondria.

### 4.18 Electron Microscopy

Tumor tissue specimens were initially immersed and fixed in a solution containing 2.5% glutaraldehyde and 1% osmium tetroxide. They were then sequentially dehydrated using a graded series of ethanol solutions. After dehydration, the specimens were embedded in an epoxy resin. Ultrathin sections were prepared for microscopic examination and stained with uranyl acetate and lead citrate. Finally, a transmission electron microscope (Hitachi Ltd., Tokyo, Japan) was used to obtain images.

### 4.19 RNA Sequencing

The NEBNext Ultra RNA Library Prep Kit for Illumina (New England BioLabs) was used to process RNA‐seq data. Briefly, TRIzol reagent was used to separate the total RNA from either control or SHP1‐depleted HCT116 cells. After purification using the PolyTtract mRNA Isolation System, poly(A) RNA was used to create cDNA libraries. The Illumina HiSeq X Ten platform was used to sequence the samples. The Illumina sequence analysis pipeline was used to align sequence reads with the human hg38 genome. The subsequent study was conducted using the average gene expression levels from three separate experiments.

### 4.20 MeRIP‐Seq

MeRIP‐seq was performed by Novogene Co., Ltd. (Beijing, China). HCT116 shNC and shSHP1 cells were isolated. Then, DNase I (Roche, Indianapolis, IN, USA) was used to treat the total RNA after extraction using TRIzol. After the RNA was disrupted into approximately 200‐nucleotide fragments, an anti‐m6A antibody (Merck Millipore) was added immediately. ChIP‐grade protein A/G magnetic beads (Merck Millipore) were added to the complexes, and a low/high salt washing technique was used to wash the complexes. Protein A/G magnetic beads were used to elute the m6A‐enriched RNA, which was then purified using an RNeasy mini kit (QIAGEN) and sequenced on an Illumina PE150 platform.

### 4.21 Lipid Nanoparticles

LNPs were assembled using Ruixi Biological Technology (Xian, China). siRNAs were purchased from Sangon Biotech (Shanghai, China). The sequences of siRNAs were as follows: human SHP1‐1 sense, GGAUUUCUAUGACCUGUAU; antisense, AUACAGGUCAUAGAAAUCC; human SHP1‐2 sense, GAGGCGCAGUACAAGUUCA; antisense, UGAACUUGUACUGCGCCUC. When tumors reached 50–100 mm^3^, the mice were randomized (*N* = 5) and treated with LNP–siNC, LNP–siSHP1‐1, and LNP–siSHP1‐2 via tail vein injection (2 mg/kg) twice per week.

### 4.22 Bioinformatics Analysis

The mRNA expression profiles of *SHP1* in CRC and normal tissues and associated clinical patient data were retrieved from TCGA and Genotype‐Tissue Expression (GTEx) project databases. Additionally, we procured gene expression data from the GSE17537 dataset in the Gene Expression Omnibus repository. To assess the correlation between *SHP1* mRNA levels and patient survival, a Kaplan–Meier survival analysis was performed. Differences in *SHP1* expression in patients, sorted according to clinical parameters, were evaluated to elucidate its effects on cancer progression. Differential gene expression was determined utilizing the “DESeq2” R package, with a threshold of a |logFC| > 1 and an adjusted *p* value < 0.05 to define significantly DEGs. Subsequent functional enrichment analyses, including GO and Kyoto Encyclopedia of Genes and Genomes pathway analyses, were performed using the “clusterProfiler” package, applying a significance cutoff of a *p* value < 0.05. The consensus phosphorylation sites were predicted using NetPhos (http://www.cbs.dtu.dk/services/NetPhos/), and the UniProt human proteome database (https://www.uniprot.org/) was used to retrieve the FTO (Q9C0B1) protein sequence, which was then submitted for online prediction.

### 4.23 Statistical Analyses

All analyses were performed in R 4.0.4. Correlation analysis was computed by the Spearman method. The Kaplan–Meier method was used, and the log‐rank test was applied for comparisons of survival. For two‐group comparisons, Student's *t* test was applied if the data were normally distributed and the Wilcoxon test was applied if the data were non‐normally distributed. One‐way analysis of variance (ANOVA) or two‐way ANOVA with Tukey's post hoc test if the group had magnitude change were used for comparisons of multiple groups. Statistical significance was defined as *p* value < 0.05. Statistical significance was demonstrated as *p < 0.05, **p < 0.01, and ***p < 0.001.

## Author Contributions

XW and CFL designed the study and finished the experiments. QYL performed the bioinformatics analysis. HQ, XLY, and CFL provided administrative support. KZ, WHZ, MY, and SBY analyzed data. LM and BL modified the manuscript. All the authors have read and approved the final manuscript.

## Conflicts of Interest

The authors declare no conflicts of interest.

## Ethics Statement

All animal experiments were approved by the Institutional Animal Care and Use Committee of Huazhong University of Science and Technology (Approve No. TJH‐201910007). The Tongji Hospital Ethics Committee authorized this research.

## Supporting information




**Supporting File 1**: mco271140‐sup‐0001‐SuppMat.pdf

## Data Availability

All the data can be obtained by contacting the corresponding author.
