# Supplementary material for: SHP1‐Mediated FTO Dephosphorylation Regulates Mitophagy and Promotes Tumor Progression via N(6)‐Methyladenosine in Colorectal Cancer
Source: MedComm (2020). 2026 Nov 8;7(11):e71140. doi: 10.1002/mco2.71140 (PMC13744649; doi:10.1002/mco2.71140)
Supplement: Supplementary file 1 [file MCO2-7-e71140-s001.pdf]

SHP1-mediated FTO dephosphorylation regulates mitophagy and promotes tumor progression via N(6)-methyladenosine in colorectal cancer

Running title: SHP1/FTO/m6A axis mediates mitophagy and cancer progression

Xi Wang[1, †], Qinyu Li[1, †], Kai Zeng[2], Weiheng Zhao[1], Mu Yang[1], Sibi Yin[1], Li Ma[1], Bo Liu[1], Hong Qiu[1, *], Chaofan Liu[1, *], Xianglin Yuan[1, *],

[1]Department of Oncology, Tongji Hospital, Tongji Medical College, Huazhong University of Science and Technology, Wuhan 430030, Hubei, China

[2]Department of Urology, the First Affiliated Hospital of Shihezi University, Shihezi, Xinjiang, China

† These authors contributed equally to this work and shared first authorship.

Corresponding to:

Professor Xianglin Yuan, Department of Oncology, Tongji Hospital, Tongji Medical College, Huazhong University of Science and Technology, Hubei Province, China, 430030. yuanxianglin@hust.edu.cn

Doctor Chaofan Liu, Department of Oncology, Tongji Hospital, Tongji Medical College, Huazhong University of Science and Technology, Hubei Province, China, 430030. lcf20101995@sina.com

Professor Hong Qiu Department of Oncology, Tongji Hospital, Tongji Medical College, Huazhong University of Science and Technology, Hubei Province, China, 430030. qiuhong@hust.edu.cn

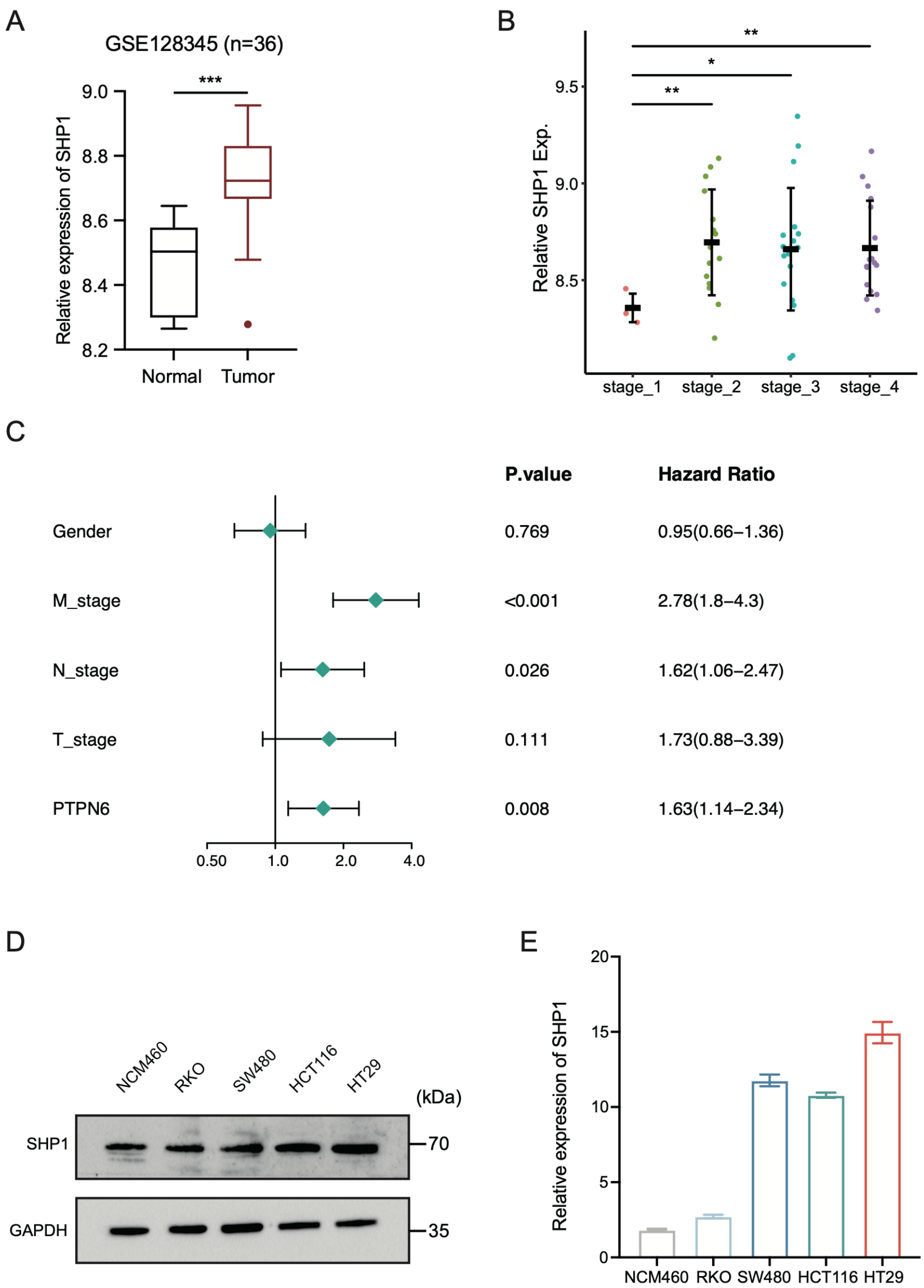

Figure S1. (A) Differential expression of PTPN6 in tumors and normal tissues based on GSE128345. (B) Differential expression of PTPN6 in patients based on different T stages, based on GSE17537. (C) Forest plot showing multivariate Cox regression analysis of SHP1/PTPN6 expression and clinicopathological variables, including gender, T stage, N stage, and M stage. (D, E) SHP1 content, measured via western blotting and qPCR, showing a significant increase in SHP1 levels in SW480, HCT116, and HT29 cells.

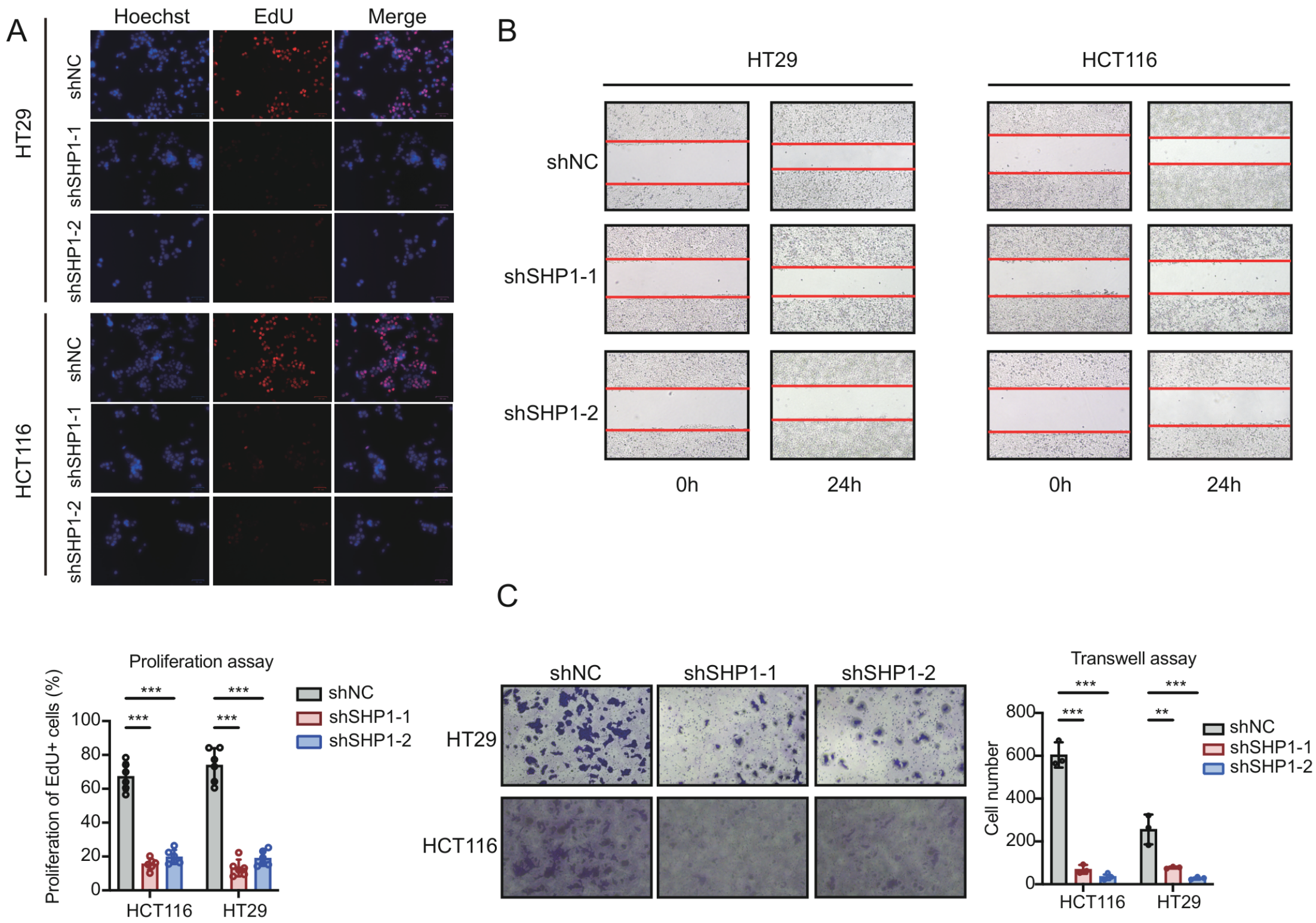

Figure S2. (A) Representative EdU staining images and quantification of HT29 and HCT116 cells after SHP1 knockdown. Hoechst was used for nuclear counterstaining. (B) Representative wound healing images of HT29 and HCT116 cells expressing shNC, shSHP1-1, or shSHP1-2 at 0 h and 24 h. (C) Representative Transwell invasion images and quantification of invaded HT29 and HCT116 cells expressing shNC, shSHP1-1, or shSHP1-2. **$p < 0.01$, ***$p < 0.001$.

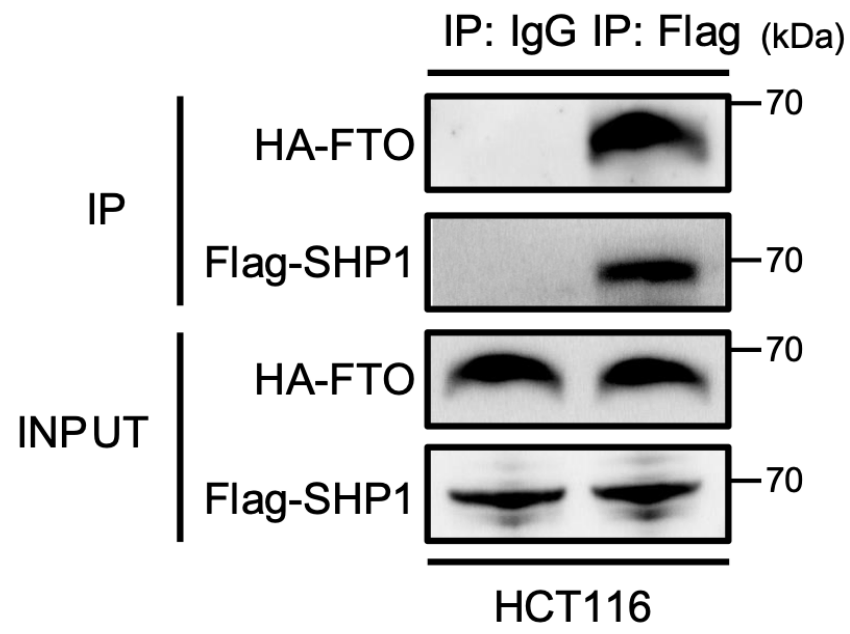

Figure S3. In vitro binding assay using recombinant proteins of SHP1 and FTO.

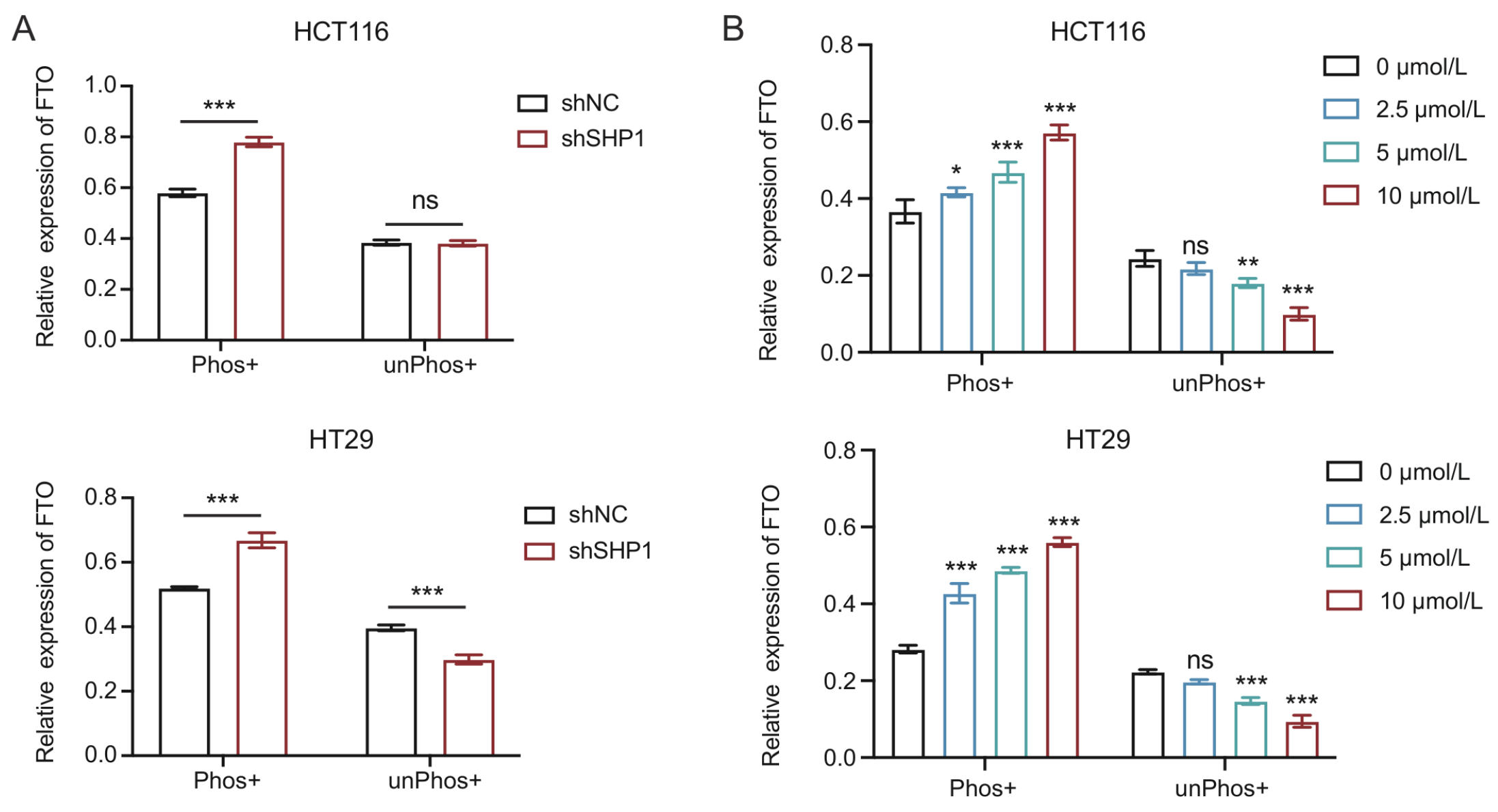

Figure S4. (A) Quantification of phosphorylated and unphosphorylated FTO protein levels in HCT116 and HT29 cells following SHP1 inhibition, relative to Figure 3D-E. (B) Quantification of phosphorylated and unphosphorylated FTO protein levels in HCT116 and HT29 cells treated with different doses of TPI-1, relative to Figure 3F-G. ns, not significant.

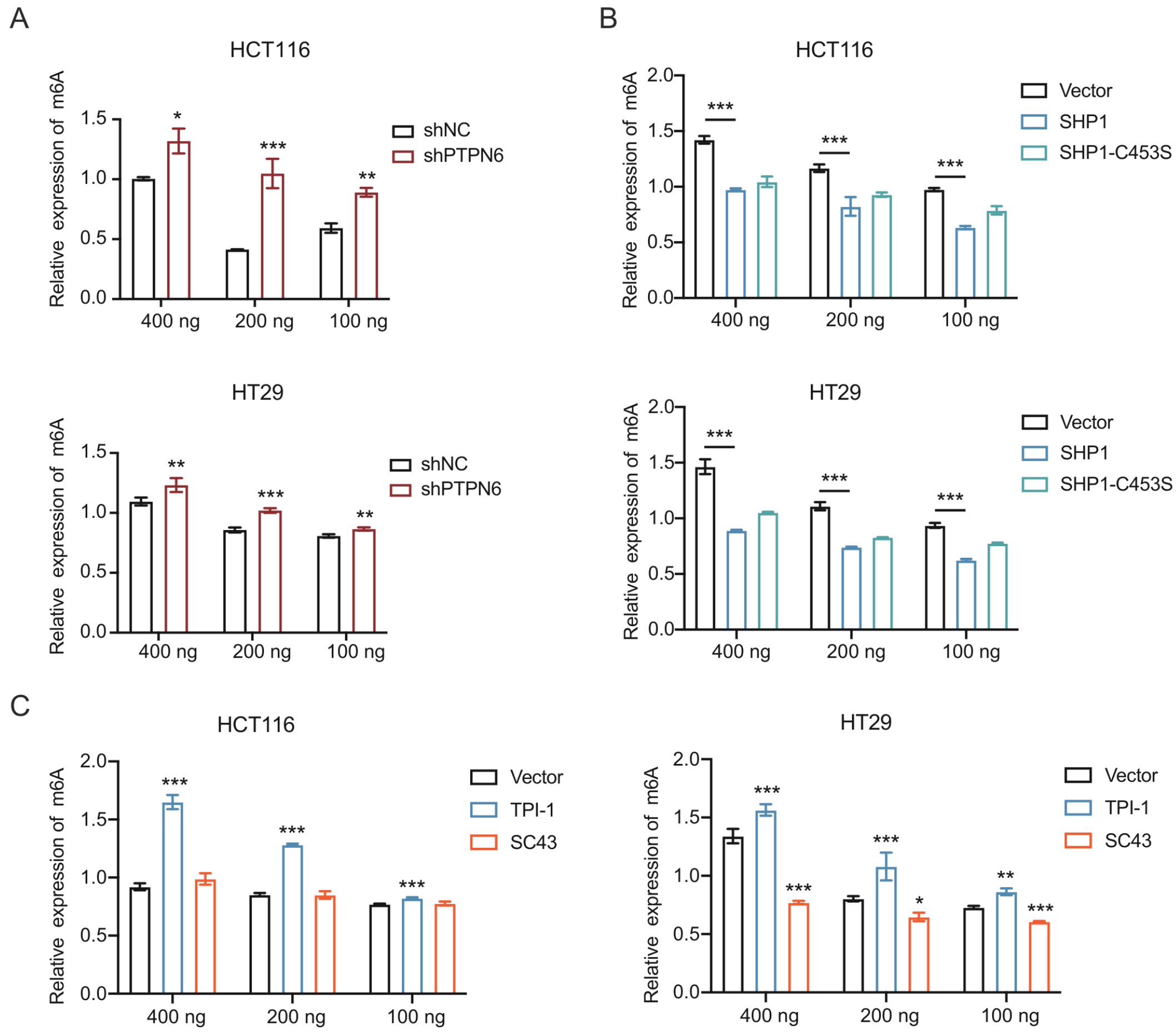

Figure S5. (A) Quantification of the relative m6A expression in HCT116 and HT29 cells after SHP1 inhibition, relative to Figure 3K. (B) Quantification of the relative expression of m6A in HCT116 and HT29 cells following the overexpression of SHP1 or SHP1-C453S, relative to Figure 3L. (C) Quantification of relative m6A expression in HCT116 and HT29 cells treated with TPI-1 or SC43, relative to Figure 3M.

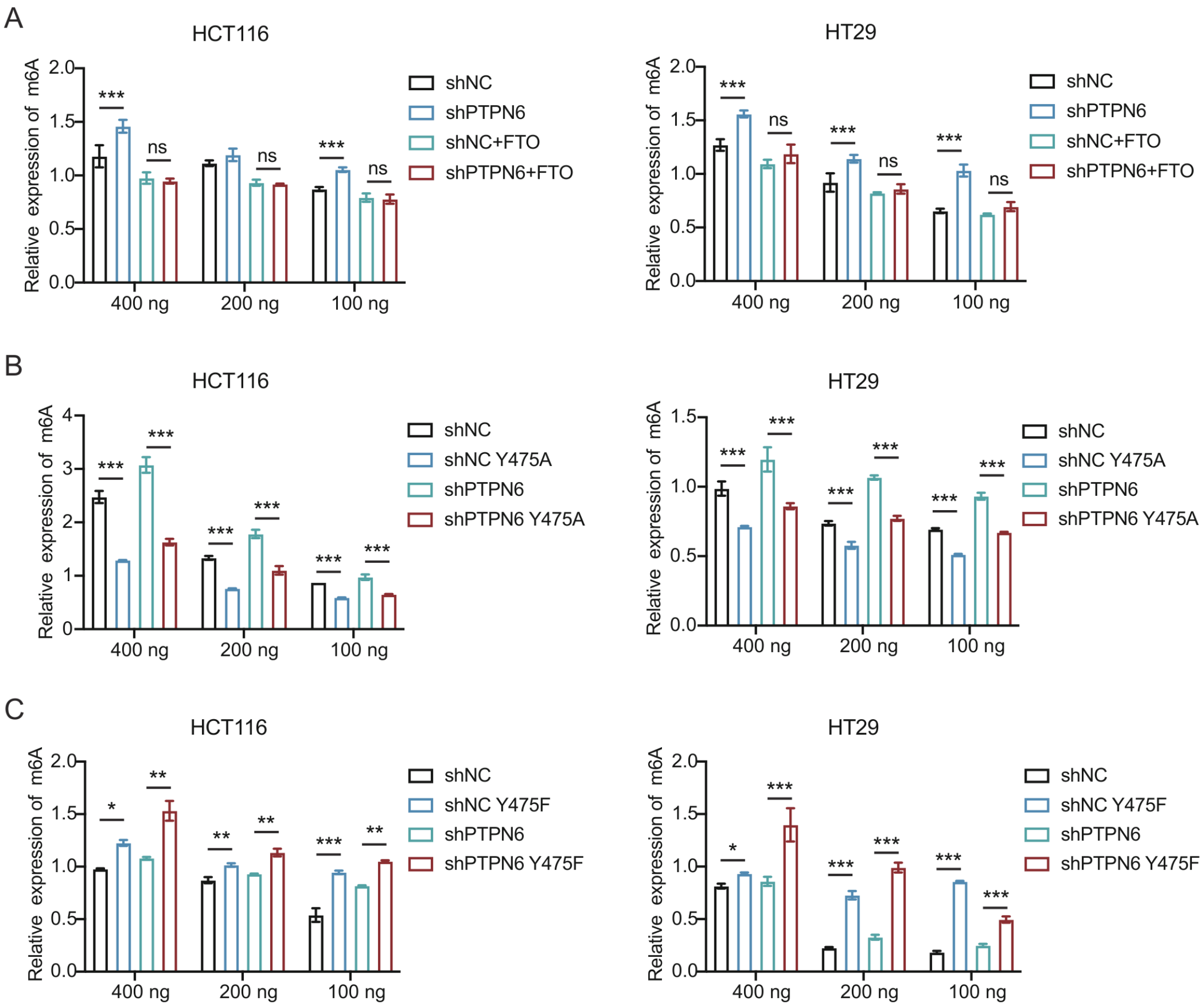

Figure S6. (A) Quantification of relative m6A expression in HCT116 and HT29 cells following SHP1 inhibition, FTO overexpression, and SHP1 inhibition with FTO overexpression. The figure is relative to Figure 3N. (B) Quantification of the relative expression of m6A in HCT116 and HT29 cells following FTO-Y475A overexpression, SHP1 inhibition, or SHP1 inhibition with FTO-Y475A overexpression. The figure is relative to Figure 3O. (C) Quantification of the relative expression of m6A in HCT116 and HT29 cells following FTO-Y475F overexpression, SHP1 inhibition, and SHP1 inhibition with FTO-Y475F overexpression. The figure is relative to Figure 3P. ns, not significant.

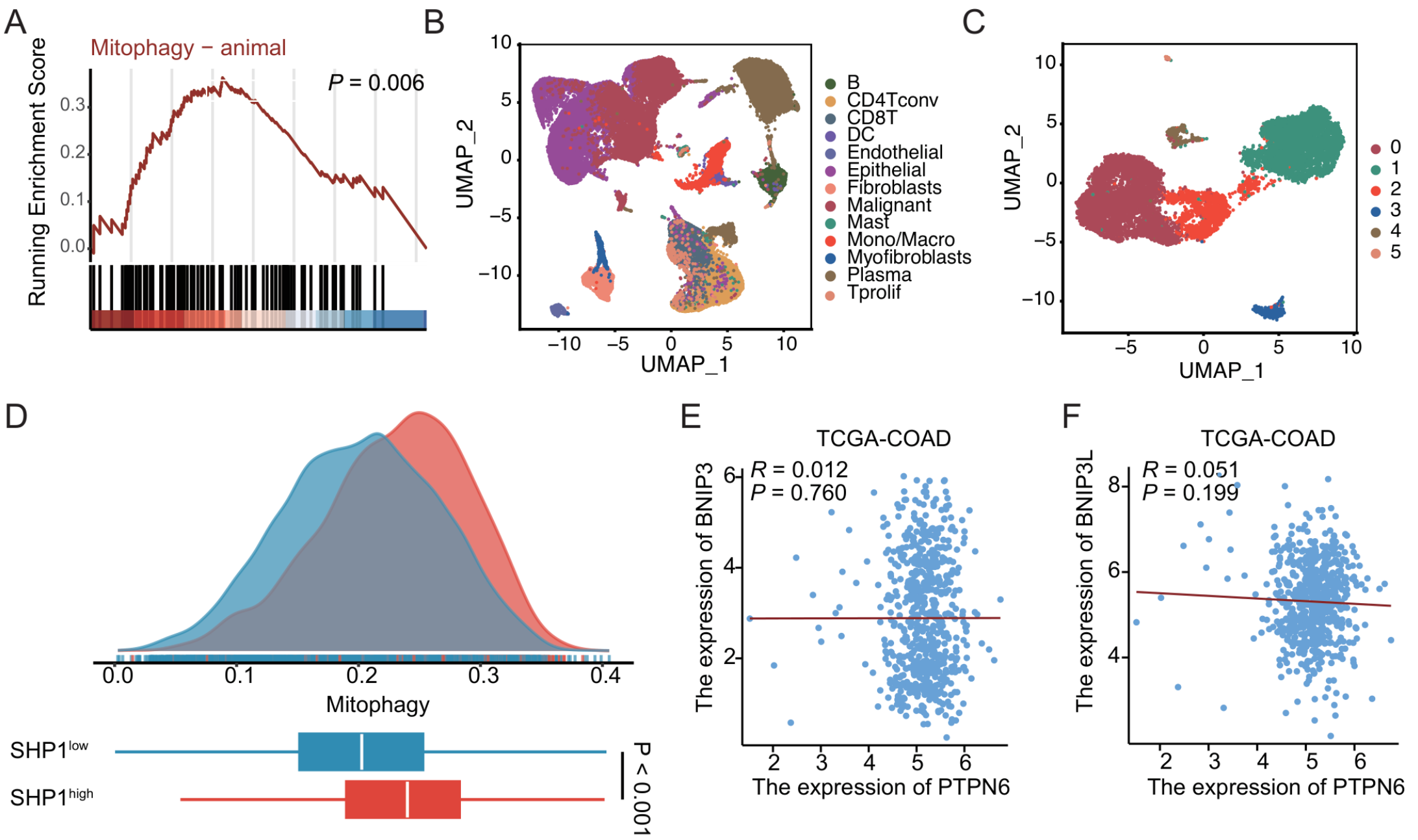

Figure S7. (A) GSEA was performed to identify significantly enriched pathways in the SHP1^{high} groups in TCGA-COAD cohort. (B) UMAP plot showing the distribution of the cell types in GSE166555. (C) UMAP plot showing the distribution of the cluster of tumor cells. (D) the mitophagy pathway was compared between the SHP1-high and SHP1-low groups at the single cell level. (E-F) Correlation analysis of SHP1 and BNIP3, BNIP3L expression in TCGA-COAD data. UMAP, uniform manifold approximation and projection; CD4Tconv, conventional CD4-positive T cells; CD8T, CD8-positive T cells; DC, dendritic cells; Mono/Macro, monocytes/macrophages; Tprolif, proliferating T cells.

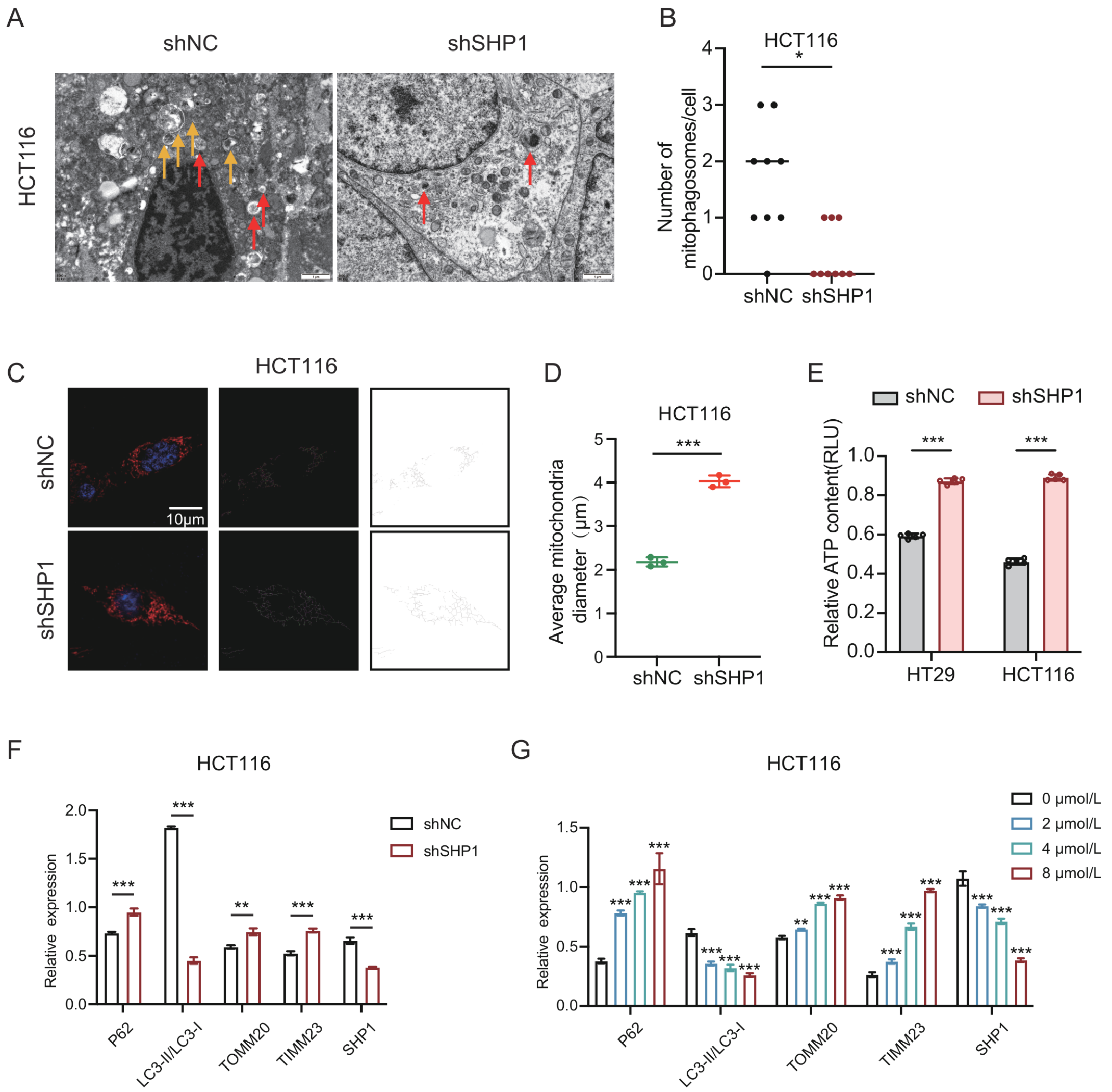

Figure S8. (A) Transmission electron microscopy analysis of xenograft tumor samples derived from HCT116 cells with SHP1 knockdown showing reduced mitophagy in vivo. (B) Quantification of mitophagy levels based on TEM images. (C) PK-Mito-Red immunofluorescence of HCT116 shNC and HCT116 shSHP1 cells. (D) Quantification of mitochondria diameter from PK-Mito-Red immunofluorescence images. (E) Relative ATP content in HT29 and HCT116 cells with or without SHP1 knockdown. (F) Quantification of relative expression of p62, LC3-II/LC3-I, TOMM20, TIMM23, and PTPN6 in HCT116 cells following SHP1 inhibition, relative to Figure 7N. (G) Quantification of relative expression of p62, LC3-II/LC3-I, TOMM20, TIMM23, and

PTPN6 in HCT116 cells treated with different doses of TPI-1. ATP, adenosine triphosphate; RLU, relative light units.

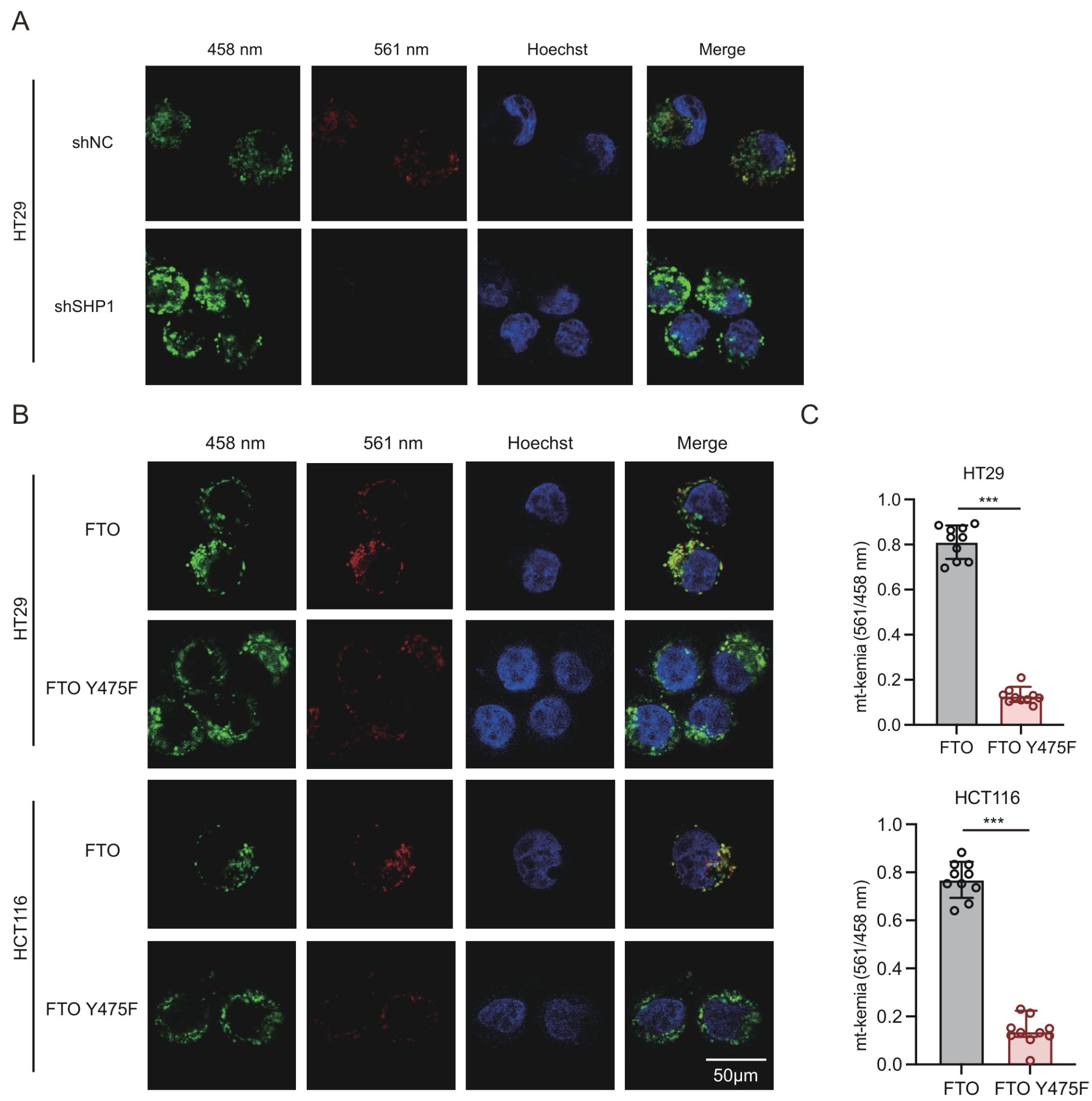

Figure S9. (A) Representative mt-Keima images showing mitophagy flux in control and SHP1-depleted HT29 cells. (B) Representative mt-Keima images of HT29 and HCT116 cells expressing wild-type FTO or FTO-Y475F. (C) Quantification of mt-Keima fluorescence ratios in the indicated cells.

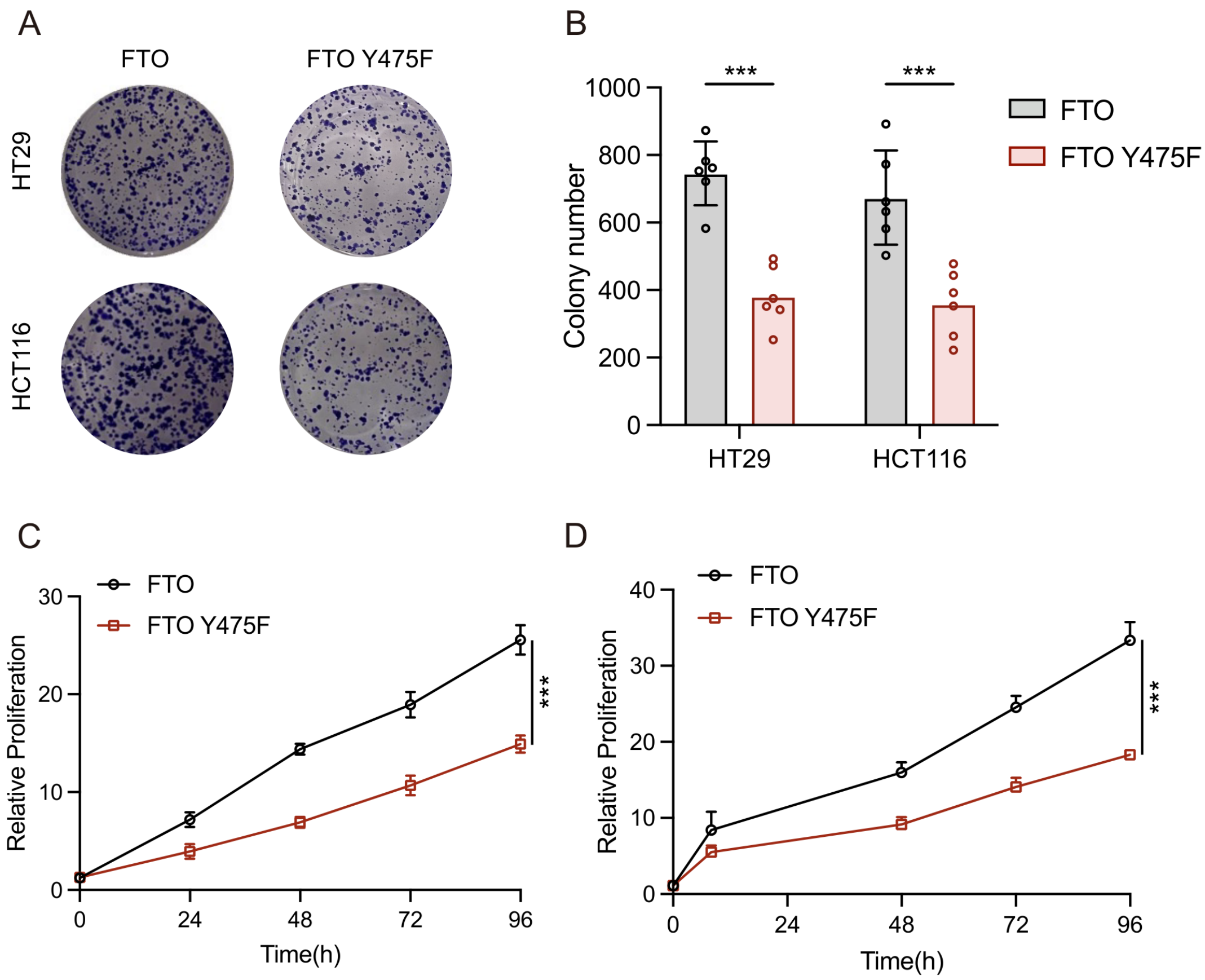

Figure S10. (A) Representative colony formation images of HT29 and HCT116 cells expressing wild-type FTO or FTO-Y475F. (B) Quantification of colony numbers in the indicated cells. (C, D) CCK-8 assays showing the proliferation of HT29 (C) and HCT116 (D) cells expressing wild-type FTO or FTO-Y475F.

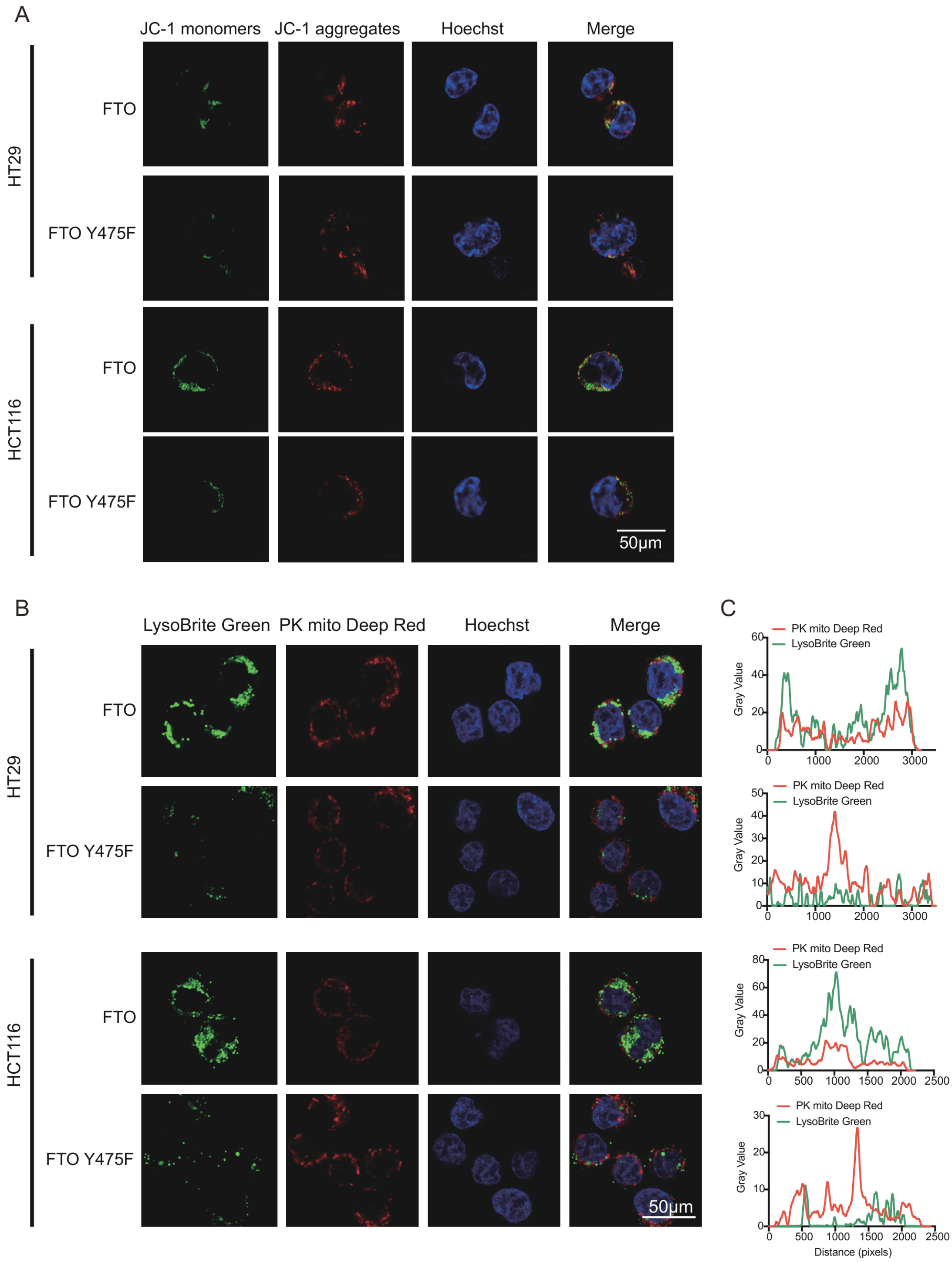

Figure S11. (A) Representative JC-1 staining images of HT29 and HCT116 cells expressing wild-type FTO or FTO-Y475F. (B-C) Representative immunofluorescence images showing lysosome-mitochondria colocalization.

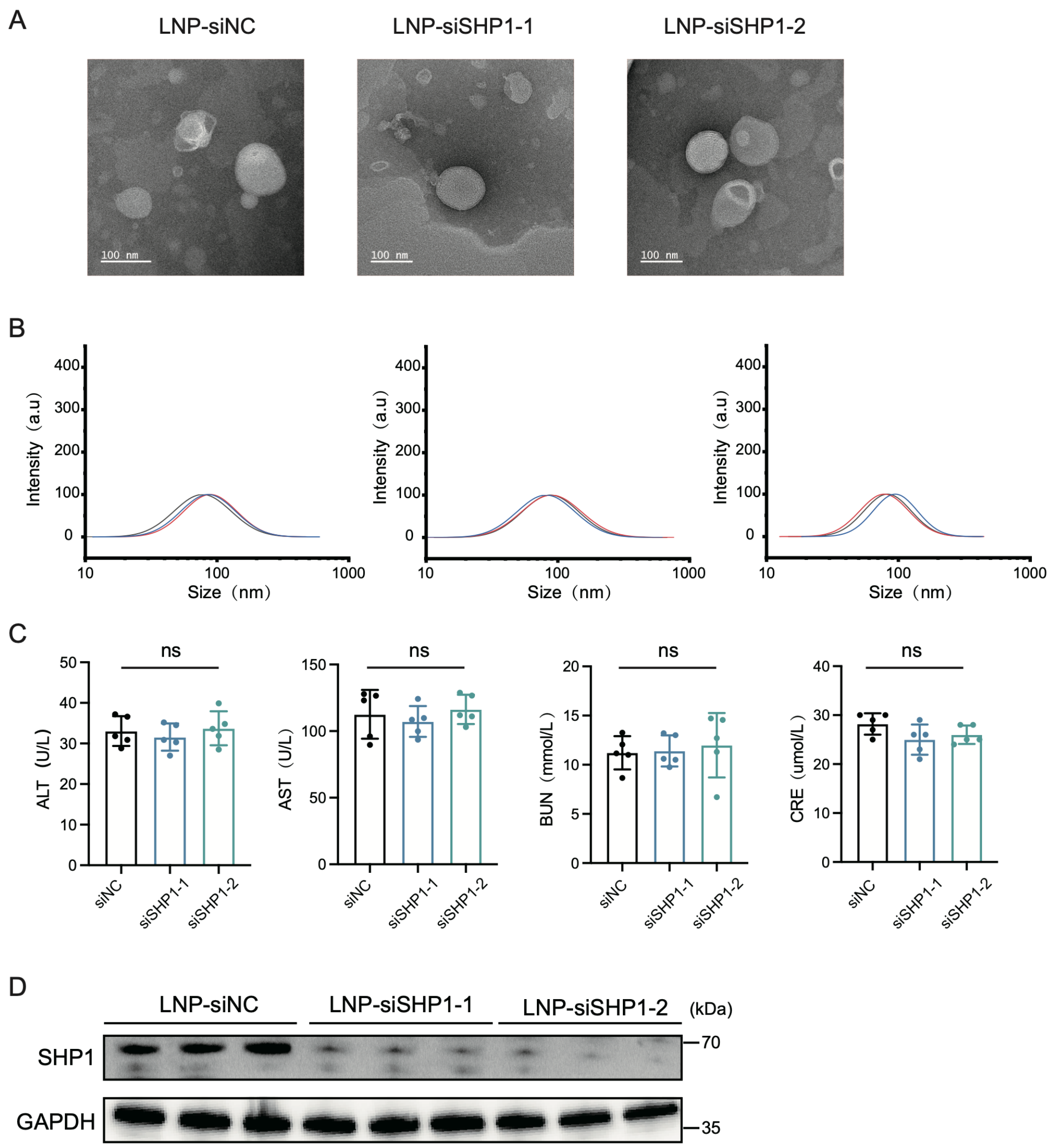

Figure S12. Characteristics of the LNP-siRNA. (A) Representative TEM images of LNP-siRNA. (B) Particle size intensity distribution curve of LNP-siRNA. (C) Systemic administration of LNP-siRNA to mice did not induce significant changes in renal functional markers (blood urea nitrogen [BUN] and serum creatinine [CRE]) or hepatic transaminase levels (serum alanine aminotransferase [ALT] and aspartate aminotransferase [AST]). All evaluated biochemical parameters maintained values within the established physiological reference ranges. (D) Western blot analysis of SHP1 protein levels in xenograft tumor tissues from LNP-siNC, LNP-siSHP1-1, and LNP-siSHP1-2 groups. a.u., arbitrary units.

Table S1. Clinical information of HColA160Su02

No. of patients	N = 100
**T stage**	
T2	3(3%)
T3	29(29%)
T3-4	1(1%)
T4a	43(43%)
T4b	19(19%)
NA	5(5%)
**N stage**	
N0	58(58%)
N1a	10(10%)
N1b	16(16%)
N1c	1(1%)
N2a	8(8%)
N2b	4(4%)
NA	3(3%)
**M stage**	
M0	99(99%)
M1	1(1%)

NA, not available.

Table S2. RNAi sequences used in human *SHP1* knockdown.

**RNAi**	**Sequence (5'-3')**
SHP1-RNAi-1	GGTGAATGCGGCTGACATTGA
SHP1-RNAi-2	GGATTTCTATGACCTGTATGG

RNAi, RNA interference.

Table S3. Primary antibodies used in IHC, IF, IB, IP and DB.

Antibody	Supplier	Catalog (clone)	Application	Dilution
FTO	Abcam	Ab126605	IB/IP	1:1000/1:100
SHP1	Abcam	ab227503	IB/IP/IHC	1:1000/1:100/1:100
Pan-Phospho-Tyrosine Rabbit mAb	Abclonal	AP1162	IB	1:1000
m6A	Abcam	ab208577	DB	1:2000
GAPDH	Proteintech	60004-1-Ig	IB	1:10,000
FITC, goat anti-rabbit IgG	Abclonal	AS011	IF	1:500
STAT3	Abclonal	A19566	IB	1:1000
SQSTM1/p62	Proteintech	66184-1-Ig	IB	1:1000
LC3A/B	Cell Signaling Technology	4108	IB/IF	1:1000/1:100
TOMM20	Abcam	ab186735	IB	1:1000
TIMM23	Abcam	ab230253	IB	1:1000
PINK1	Abcam	ab216144	IB	1:1000
Rabbit anti-HA	Cell Signaling Technology	4793	IB	1:1000
Flag	Sigma	F1804	IB/IP	1:1000/1:100
HRP, goat anti-rabbit IgG LCS	Abbkine	A25012	IB	1:10,000
HRP-conjugated goat anti-rabbit IgG	Abclonal	AS014	IB	1:10,000
HRP-conjugated goat anti-mouse IgG	Abclonal	AS003	IB	1:10,000

Abbreviations: IB, immunoblotting; IP, immunoprecipitation; DB, Dot Blotting; IF,

immunofluorescence staining; IHC, immunohistochemistry staining; FTO, fat mass and obesity-associated protein; SHP1, Src homology region 2 domain-containing phosphatase 1; STAT3, signal transducer and activator of transcription 3; SQSTM1/p62, sequestosome 1; LC3A/B, Microtubule Associated Protein 1 Light Chain 3 Alpha/Beta; TOMM20, Translocase Of Outer Mitochondrial Membrane 20; TIMM23, Translocase Of Inner Mitochondrial Membrane 23; PINK1, PTEN Induced Kinase 1; mAb, monoclonal antibody; HRP, horseradish peroxidase; LCS, light chain-specific.

Table S4. Primer sequences used in RT-qPCR.

Primer	Sequence (5'-3')
Human *PTPN6* forward	GGTGTCCACGGTAGCTTCC
Human *PTPN6* reverse	ACAGGTCATAGAAATCCCCTGAG
Human *PINK1* forward	GCCTCATCGAGGAAAAACAGG
Human *PINK1* reverse	GTCTCGTGTCCAACGGGTC
Human *GAPDH* forward	GGAGCGAGATCCCTCCAAAAT
Human *GAPDH* reverse	GGCTGTTGTCATACTTCTCATGG

Table S5.RNA sequence of m6A

PINK1-m6A	caagUccUgUcacaUaaA(m6A)ccaggUggUcUaagcagA(m6A)ccccUUcUgggUcUgag
PINK1-NC	caagUccUgUcacaUaaAccaggUggUcUaagcagAccccUUcUgggUcUgag
